# Geographic Authentication of *Eucommia ulmoides* Leaves Using Multivariate Analysis and Preliminary Study on the Compositional Response to Environment

**DOI:** 10.3389/fpls.2020.00079

**Published:** 2020-02-19

**Authors:** Chao-Yong Wang, Li Tang, Li Li, Qiang Zhou, You-Ji Li, Jing Li, Yuan-Zhong Wang

**Affiliations:** ^1^National & Local United Engineering Laboratory of Integrative Utilization Technology of Eucommia Ulmoides, Jishou University, Jishou, China; ^2^College of Biological Resources and Environmental Sciences, Jishou University, Jishou, China; ^3^College of A & F Science and Technology, Hunan Applied Technology University, Changde, China; ^4^College of Chemistry and Chemical Engineering, Jishou University, Jishou, China; ^5^Institute of Medicinal Plants, Yunnan Academy of Agricultural Sciences, Kunming, China

**Keywords:** *Eucommia ulmoides* leaves, Fourier Transform Near-Infrared, Attenuated Total Reflection Fourier Transform Mid-Infrared, chemometrics, environment

## Abstract

To explore the influences of different cultivated areas on the chemical profiles of *Eucommia ulmoides* leaves (EUL) and rapidly authenticate its geographical origins, 187 samples from 13 provinces in China were systematically investigated using three data fusion strategies (low, mid, and high level) combined with two discrimination model algorithms (partial least squares discrimination analysis; random forest, RF). RF models constructed by high-level data fusion with different modes of different spectral data (Fourier transform near-infrared spectrum and attenuated total reflection Fourier transform mid-infrared spectrum) were most suitable for identifying EULs from different geographical origins. The accuracy rates of calibration and validation set were 92.86% and 93.44%, respectively. In addition, climate parameters were systematically investigated the cluster difference in our study. Some interesting and novel information could be found from the clustering tree diagram of hierarchical cluster analysis. The Xinjiang Autonomous Region (Region 5) located in the high latitude area was the only region in the middle temperate zone of all sample collection areas in which the samples belonged to an individual class no matter their distance in the tree diagram. The samples were from a relatively high elevation in the Shennongjia Forest District in Hubei Province (>1200 m), which is the main difference from the samples from Xiangyang City (78 m). Thus, the sample clusters from region 9 are different from the sample clusters from other regions. The results would provide a reference for further research to those samples from the special cluster.

## Introduction

*Eucommia ulmoides* Oliver is the single species of the genus *Eucommia*, which in turn is the only genus in the family Eucommiaceae. This plant has been applied as a tonic herb in China since ancient times (Dai et al., 2013). According to geographical and historical investigations, the plant is widely distributed in Asia (mainly cultivated in China), Europe, and North America (Hirata et al., 2014). It is also known as Du-Zhong (in Chinese) and Tuchong (in Korean and Japanese) (Do et al., 2018; Zhang et al., 2018). Various compounds were extracted and identified from each part of *E. ulmoides*. These include lignans, iridoids, flavonoids, phenols, steroids, and terpenes and nutrients, i.e., amino acids, vitamins, and mineral elements (Hussain et al., 2016; Wang et al., 2019). In modern pharmacological studies, researchers have proved that chemical profiles possess encouraging medical curing effects on hypertension, hyperglycemia, hyperlipidemia, osteoporosis, osteoarthritis, antioxidant, etc. (Luo et al., 2010; Xie et al., 2015; Niu et al., 2016; Wang et al., 2019). The medicinal parts of *E. ulmoides* are its barks (Eucommiae Cortex) and leaves (Eucommiae Folium) as mentioned in the Chinese Pharmacopoeia (Chinese Pharmacopoeia Commission, 2015). Barks are not conducive to large-scale development and utilization due to their resource constraints, but can be used in small amounts for disease treatment. Up to now, scientific research has allowed the identification of chemical constituents, and the pharmaceutical functions in the leaves of *E. ulmoides* have been found to be similar to those in the bark.

Compared with barks, EULs can be harvested every year. The developed orchard cultivation mode greatly facilitated the harvesting of EULs (Zhu et al., 2016). In China, EULs have been included in the management of “Affinal Drug and Diet”. EUL extract can be used in the production of functional foods and various beverages. Moreover, it can be used to separate and extract active ingredients for drug manufacturing. In addition to the above applications, EUL extract are used as a feed additive for antibiotic-free breeding. EULs are adopted as a folk remedy for the treatment of diabetes in Korea (Hong et al., 1987). The commercial product (Tochu-cha in Japanese) is a government-approved food for specified healthcare for people with hypertension (Hosoo et al., 2017). Moreover, EUL residues that remain after extracting the effective active components are used as a raw material for gutta-percha extraction. The glued residues can be further used to manufacture a variety of products, such as sheets, organic fertilizers, and fuel. Such method reduces the production cost of gutta-percha and establishes an important foundation for the large-scale production of gutta-percha. In particular, the abundant chemical profiles of EUL are the basis for industrial development and application. As a raw material for comprehensive utilization, the quality evaluation of EUL is particularly important.

Conventionally, high (ultra) performance liquid chromatography coupled with diode array detector or mass spectrometry is the common tool for analyzing of chemical composition and for determination of EUL’s relative content (Niu et al., 2016; Li et al., 2017; Yan et al., 2018). Essential oil and their chemical constituent can be examined through gas chromatography linked with flame ionization or mass spectrometry (Farag et al., 2018; Kfoury et al., 2018). However, these methods normally require deleterious and dangerous reagents, such as methanol and acetonitrile. Moreover, the sample solution preparation process for chromatographic analysis is cumbersome, and the instrument operation requirements are high. In order to obtain a more simple and convenient method for quality evaluation of EULs, fast and non-destructive spectroscopy technologies, including vibrational spectroscopy, have become particularly attractive because of their unique advantages in terms of cost, efficiency, sample preparation, and instrumentation (Ma et al., 2018). In addition, the spectra can reflect the entire chemical information of a sample, rather than the determination and characterization of a single component in the liquid chromatograms. However, compared with liquid chromatography, infrared technology is limited, because it cannot be accurately quantified, and the chemical information reflected is ambiguous. To date, vibrational spectroscopic techniques, including Fourier transform (FT) mid-infrared (MIR), near-infrared (NIR) spectroscopy, and Raman spectroscopy, have been widely used in food production (Shi and Yu, 2017; Qi et al., 2018) and in the pharmaceutical (Jamrógiewicz, 2012; Li et al., 2018) and agriculture (Yang and Ying, 2011; Horn et al., 2018) industries. These methods also exhibit great potential for application to disease status monitoring (Depciuch et al., 2017; Kaznowska et al., 2018).

The composition of secondary metabolites in plant tissues varies depending on different factors, such as genes, climate, altitude, and growth environment conditions in general (Ložienė and Venskutonis, 2005; Shafie et al., 2009; Zheng et al., 2012; Zheng et al., 2018). Li and Wang (2018a) investigated the chemical information of the two medicinal parts (epidermis and inner part) of *Wolfiporia cocos*; the inner parts had better quality consistency, which was affected by the main factor, i.e., the epidermis’ poor resistance to the external environment. Uleberg et al. (2016) suggested that the production and quality of berries were affected by climate, particularly when berries lived in a challenge northern climate with low winter temperatures and long days through the growing season. Zheng et al. (2011) reported the correlation between latitude and altitude and the values of sugars, sugar alcohols, ascorbic acid, and fruit acids in wild *Hippophaë rhamnoides*. The strong adaptive capability to a changeable environment condition makes *E. ulmoides* a widespread species in China, with large scope latitude (N24.5°–N41.5°) and longitude (E76°–E126°) and high drop altitude (50–2,500 m) (Du et al., 2013). Various climate types and soil types exist in these suitable areas for growth. Maintaining the quality consistency for EULs despite the fact that *E. ulmoides* plants grow in complex and varied environments is a challenge. Therefore, the geographic authentication and quality assessment for EULs is of vital importance.

We selected infrared spectroscopy (MIR and NIR) rather than other expensive techniques to examine chemical profiles for the geographical authentication of EUL samples from 13 provinces in China. A multi-spectral information fusion study of EUL has not been conducted thus far. Therefore, we intended to combine both NIR and MIR spectral information to demonstrate the feasibility of discriminate EUL geographic origins based on RF and PLS-DA algorithms and data fusion strategy and to make a preliminary evaluation of the influence of the growth environment on the accumulation of EUL chemical components. Our results can be useful for determining the geographical traceability of EUL products in the market and have potential broader applications to the management of the safety tracking of food or health products.

## Materials and Methods

### Samples Information

A total of 187 specimens of EULs were collected from 13 provinces of China (23 different sites with varied growth environments) from 25 May 2017 to 10 June in 2017 (**Table 1**). These leaves were collected from the central part of the canopy of the plant, including the sunny and shady slopes. This way, we avoid the impact of individual differences in samples due to collection from different parts (upper, middle and lower parts of the canopy). All of the samples were authenticated by Professor Ke-Gang Li, and voucher specimens (JIUDZ2017001-JIUDZ2017023) were deposited to the specimen repository of the Herbarium of Jishou University (JIU) in Hunan Province. EULs were dried in an oven (Experimental Instrument Factory, Shanghai, China) at 40°C until constant weight was achieved. Dried EULs were separately ground into a fine powder and passed through an 80-mesh sieve. Finally, the processed powders were stored at room temperature and kept away from direct sunlight until the next measurement.

**Table 1:** Information of the leaves (EUL) samples.

Region	Number of individuals	Collection site	Latitude (N)	Longitude (E)	Elevation (m)
1	11	Pingxiang City, Jiangxi Province	27°41′55.96″	114°05′49.77″	150
2	31	Zunyi City, Guizhou Province	27°24′04.63″	106°57′42.17″	857
		Zunyi City, Guizhou Province	27°43′29.82″	106°52′49.39″	946
		Zunyi City, Guizhou Province	27°38′42.25″	106°53′35.42″	925
3	10	Guangyuan city, Sichuan Province	32°13′38.78″	106°18′06.24″	524
4	21	Ankang City, Shaanxi Province	32°54′11.78″	108°30′36.21″	443
		Hanzhong City, Shaanxi Province	33°20′02.71″	106°00′18.25″	727
5	7	Ürümqi City, Xinjiang Autonomous Region	44°02′35.60″	87°27′45.06″	205
		Fukang City, Xinjiang Autonomous Region	44°18′32.65″	88°35′38.32″	513
6	20	Zhangjiajie City, Hunan Province	29°31′22.69″	110°46′02.50″	334
		Jishou City, Hunan Province	28°18′17.38″	109°38′13.42″	285.1
7	16	Shennongjia Forestry District, Hubei Province	31°28′44.43″	110°22′43.08″	1343
		Xiangyang city, Hubei province	32°00′56.49″	112°09′59.91″	78
		Shennongjia Forestry District, Hubei Province	31°26′55.95″	110°23′89.11″	1,247
8	30	Pingdingshan City, Henan Province	34°04′21.02″	113°12′52.28″	252
		Lingbao City, Henan Province	34°16′56.56″	110°39′13.91″	984
		Xingyang City, Henan Province	34°43′10.21″	113°17′18.14″	420
9	5	Longnan City, Gansu Province	32°52′53.59″	104°24′12.91″	166
		Longnan City, Gansu Province	32°53′46.02″	104°22′57.99″	1,763
10	10	Nanjing City, Jiangsu Province	32°04′77.28″	118°45′73.63″	347
11	6	Dingzhou City, Hebei Province	38°53′00.38″	115°22′03.12″	68
12	10	Lu’an City, Anhui Province	31°28′30.79″	115°50′50.84″	266
13	10	Linzi City, Shandong Province	36°77′40.87″	118°30′63.12″	31

### Fourier Transform Near-Infrared (FT-NIR) Spectroscopy Analysis

Sample powders were scanned using an FT-NIR spectrometer (PerkinElmer, USA) equipped with a diffused reflection accessory. The detection wavenumber ranges were 10,000–4,000 cm^−1^ with a resolution of 4 cm^−1^ and 32 scans per spectra. Each collected spectrum was recorded as the logarithm of reciprocal reflectance, log (1/Reflectance). To reduce human operation error, each sample powder (1.0 ± 0.05 g) was weighed by electronic balance (Sartorius, Germany) and placed inside a uniform clean glass vessel for scanning. Prior to each scan, laboratory air (H_2_O and CO_2_) spectrum was recorded as background absorption and automatically deducted to eliminate the interference of air information. Constant conditions (25°C/30% RH) were controlled in order to maintain the consistency of the experiment’s operation environment. Each sample was measured in triplicate. The obtained spectra were then analyzed by SIMCA-P^+^ 14.1 (Umetrics, Sweden), a data processing software, and averages were obtained prior to further analysis.

### Attenuated Total Reflection Fourier Transform Mid-Infrared (ATR-FT-MIR) Spectroscopy Analysis

Fourier transform mid-infrared spectrometer (PerkinElmer, USA) with deuterated triglycine sulfate (DTGS) detector equipped with an attenuated total reflectance (ATR) mode (horizontal golden gate single reflection diamond) was used to collect the sample spectra. Each spectrum was conducted in the 4,000–650 cm^−1^ range with a resolution of 4 cm^−1^ and a total of 32 scans. A stainless-steel circular ring was placed on the reflection diamond to obtain a constant layer thickness for each determination. A pressure tower is placed on the top of the circular ring (PerkinElmer Inc. micrometric pressure device). When the sample spectrum was obtained, each sample powder was placed into the circular ring hole, after which the pressure tower was rotated to press the powder tightly until a consistent pressure (131 ± 1 bar) was obtained to achieve reproducible results. After each measurement, the surface of ATR crystal, circular ring and apex of pressure tower were individually wiped with a lint-free tissue containing a combination of alcohol and deionized water. The next sample was detected when the equipment was dry to avoiding mutual interference between the samples. The temperature and humidity of the laboratory remained the same as in the FT-NIR spectroscopy analysis. Each sample was measured in triplicate, these obtained spectra were then analyzed by SIMCA-P^+^ 14.1.

### Spectra Data Pretreatment

The collected ATR-FT-MIR spectra had to undergo advanced ATR correction and transformed transmittance into absorbance by OMNIC 9.2 (Thermo Fisher Scientific, USA). Raw spectra data contained vast noise and interference information. Different preprocessing methods were applied to optimize the dataset for systematic noise reduction and baseline correction (Zhuang et al., 2015; Wu et al., 2019). Multiplicative scatter correction (MSC) and second derivative (SD) were selected to reduce the effects of low levels of scattering and to correct the baseline drift effect of FT-NIR and ATR-FT-MIR spectra (Li et al., 2013; Wu et al., 2019). The “MSC+SD” combination was adopted to pretreat the spectra dataset. All preprocessing steps were conducted by SIMCA-P^+^ 14.1. Some spectral regions were removed prior to chemometric analysis because of the interference information (e.g., 4,000–3,700 cm^−1^, 2,799–1,800 cm^−1^ and 682–653 cm^−1^; they represented the baseline area and the absorbance of diamond crystal and CO_2_). Thus, each sample finally consisted of ~4,145 preprocessed data points (FT-NIR, ~3,098 variable numbers; ATR-FT-MIR, ~1,047 variable numbers), and data matrices were used for further chemometric analysis.

### Environmental Information Acquisition

For the exploratory analysis, geographical and climate information were used to assess possible relations of chemical accumulation. Meteorological background data set and soil types for the sampling sites of EULs were downloaded from the Resource and Environment Data Cloud Platform, Institute of Geographic Sciences and Natural Resources Research, Chinese Academy of Sciences (http://www.resdc.cn/DOI/doi.aspx?DOIid=39). Meteorological background data set was based on the meteorological data of 1,915 stations in China. After sorting and inspection, the original database was formed. It included the monthly precipitation and monthly average temperature of each site. Then, the annual average temperature, annual average precipitation, and ≥10°C accumulated temperature were calculated based on the site data. The annual average temperature, annual average precipitation, ≥10°C accumulated temperature, and moisture index (Thornthwaite method) spatial distribution data set with a spatial resolution of 500 m × 500 m are interpolated by the inverse distance weighted average method. More than 60% of China’s regions are mountainous. Thus, the meteorological indicators in mountainous areas are largely affected by the topography. The data set was corrected by a 1:100 million digital elevation model (DEM). The DEM correction was carried out for annual average precipitation and ≥10°C accumulated temperature based on the temperature decrease rate of 0.6°C for each 100 m rise in altitude. The locations of the sampling points in the meteorological background dataset and the soil type map were all drawn by using ArcGIS 10.0 (ESRI Inc., USA), and the data of the sampling points were extracted by the extraction analysis tool under the arc toolbox of ArcGIS.

### Chemometrics Analysis

#### Exploratory Analysis

Principal component analysis (PCA) transforms raw data into a set of linearly independent representations of each dimension through linear transformation, which can be used to extract the main feature components of the data and turn the high- dimension data into low dimension data. Through the extracted principal components, the data were transformed into a new coordinate system, and the correlation between the samples and the variables can be seen, thereby allowing the visual analysis of the samples’ classification trend (Pei et al., 2019).

In addition to the linear dimensionality reduction algorithm of PCA, a nonparametric and nonlinear algorithm of t-distributed stochastic neighbor embedding (t-SNE) also exists. t-SNE finds the law in the data by identifying the observed patterns based on the similarity of data points with multiple features. By reducing the tendency of gathering the points in the center of the map, this algorithm can lead to obvious and better visualizations (Maaten and Hinton, 2008).

Different from the above 2D reduction algorithms, hierarchical cluster analysis (HCA) determines the similarity between data points of each category and all data points by determining the distance between them. A small distance results in high similarity. The two closest data points or categories were combined to generate a clustering tree. Samples with similar chemical profiles were clustered into one group, whereas the samples with larger differences were divided into different groups according to the theory of hierarchical clustering algorithm (Pei et al., 2019).

By comparing the visualization results of the above mentioned three algorithms, we initially explored the differences between the effects of weather conditions and altitudes of different geographical ranges on the chemical composition of EUL. PCA and t-SNE were completed by MATLAB R2017a (Math Works, USA). HCA was performed by IBM SPSS Statistics 20.0 (IBM Corp., USA).

#### Partial Least Squares Discrimination Analysis (PLS-DA)

PLS-DA is a variant classifier of partial least squares regression algorithm. PLS-DA model shows the relationship among the variable matrices (*X*), which are used to predict which class the unknown sample belongs to. In the calculation, the observed *X* matrix was transformed into a set of several intermediate linear latent variables (LVs). The first *n* LVs were selected according to the maximum eigenvalue >1. For the establishment of PLS-DA classification models, the dataset was split into two subsets, i.e., the ratio of the calibration set and validation set was 2:1, by Kennard-Stone algorithm. At the same time, the model was constructed using a calibration set with 7-fold cross validation. The veracity of a prediction model was evaluated in terms of some statistical parameters including R^2^(X), Q^2^(Y), root mean square error of estimation (RMSEE) and root mean square error of cross validation (RMSECV). R^2^(X) indicates the cumulative interpretation ability and Q^2^(Y) indicates the prediction ability of the well-established model. Furthermore, the permutation test was calculated to validate the fitting degree of the PLS-DA model based on the results of R^2^-intercept and Q^2^-intercept. The permutation test of each category model was conducted with 20 iterations. Except for the steps of the Kennard-Stone algorithm that were calculated by MATLAB R2017a, the rest of the operation was performed on the SIMCA-P^+^ 14.1 software.

#### Random Forest (RF)

Compared with PLS-DA, RF exhibited a stronger processing ability for nonlinear high-order interaction data sets. This is also a non-parametric algorithm that is based on learning strategy (Wu et al., 2019). The RF algorithm has been successfully applied to the classification problems in food research (Amjad et al., 2018; Qi et al., 2018). However, there are no reports on the application of the RF model to *E. ulmoides* research. For this algorithm, thousands of trees are involved, and each tree was grown based on bootstrap sampling. Generally, the number of the tree and the branch need to be adjusted according to out-of-bag (OOB) error. Based on OOB error, the parameters (number of tree size-n_tree_, number of variables-m_try_) of the model needed to be optimized to enhance the performance. The values of best n_tree_ are used for selection of m_try_ on the basis of the lowest OOB error. Finally, we exported the confusion matrix, and calibrated and verified votes for each sample. For high-level data fusion, a single spectra matrix requires the creation of a new spectral matrix by the selected importance variables. The permutation accuracy importance shows a strong preference for the discontinuous variable (Li et al., 2018). Therefore, this method was applied for the variable selection. The RF models were performed by RStudio (Version 1.1.463), and the calculation process was carried out by using the random Forest package in R (Version 3.5.2).

#### Evaluation of Model Performance

The PLS-DA and RF models were optimized through related parameter adjustment, and the classification performance of each class in the model was evaluated based on the sensitivity (SEN), specificity (SPE), precious (PRE), and efficiency (EFF) of the calibration set and the validation set. The values of these four conceptions were further calculated by true positive (TP), false positive (FP), true negative (TN), and false negative (FN). TP and TN represent the correctly identified samples of positive and negative classes, respectively. On the contrary, FP and FN represent the incorrectly identified samples of positive and negative classes, respectively.

SEN=TP/(TP+FN)

SPE=TN/(TN+FP)

PRE=TP/(TP+FP)

$$\text{EFF}=\sqrt{\text{SEN}\times \text{SPE}}$$

Four parameters were calculated synergistically to evaluate of model performance. Amongst these parameters, sensitivity (true positive rate) displays the fraction of samples belonging to the defined class, which is correctly accepted by the category. Specificity indicates the true negative rate and samples not belonging to the specified class that is rejected by the modelled class. Efficiency is a summarizing parameter that concerns both sensitivity and specificity. Precision is the ratio between accepted samples in specified samples and totally accepted samples of the model in the calibration or validation set.

#### Data Fusion

To avoid the influence of different magnitudes, the dataset was normalized in the range of [−1, 1]. Data fusion techniques were used for coalescing information from different instruments to obtain a more accurate and holistic description. It could offset the deficiency among different analytical instruments (Li et al., 2017). In other words, the merging technique could improve the quality of sample chemical profiles and provide complementary information. The fusion strategy was divided into three levels, namely, low-, mid-, and high- levels, according to the form of data pre-processing (Wang et al., 2018; Wu et al., 2018).

Low-level data fusion, raw data from different instruments are directly spliced according to the sample number, and each sample achieves a new fingerprint for further analysis (Simonetti et al., 2016). However, for the mid-level data fusion strategy, the information characteristics of each instrument’s raw data are extracted by multiple feature extraction algorithms and then aligned by sample number and spliced into a single matrix for multivariate analysis (Spiteri et al., 2016; Li and Wang, 2018b). High-level data fusion was used to compare the classification results of data sets (calibration and validation sets) from different sources. The same classification categories are divided into this category, and different classification categories are based on the results of the fuzzy set theory (Márquez et al., 2016; Li et al., 2018). The result of the majority voting is the classification category. The four fuzzy connection operators through the minimum, maximum, average, and product are used to identify the inconsistent samples of the independent model and reclassify the sample. All samples are ultimately assigned to the majority vote (Márquez et al., 2016).

## Results and Discussion

### Interpretation of FT-NIR and ATR-FT-MIR Spectra

**Figure 1:**
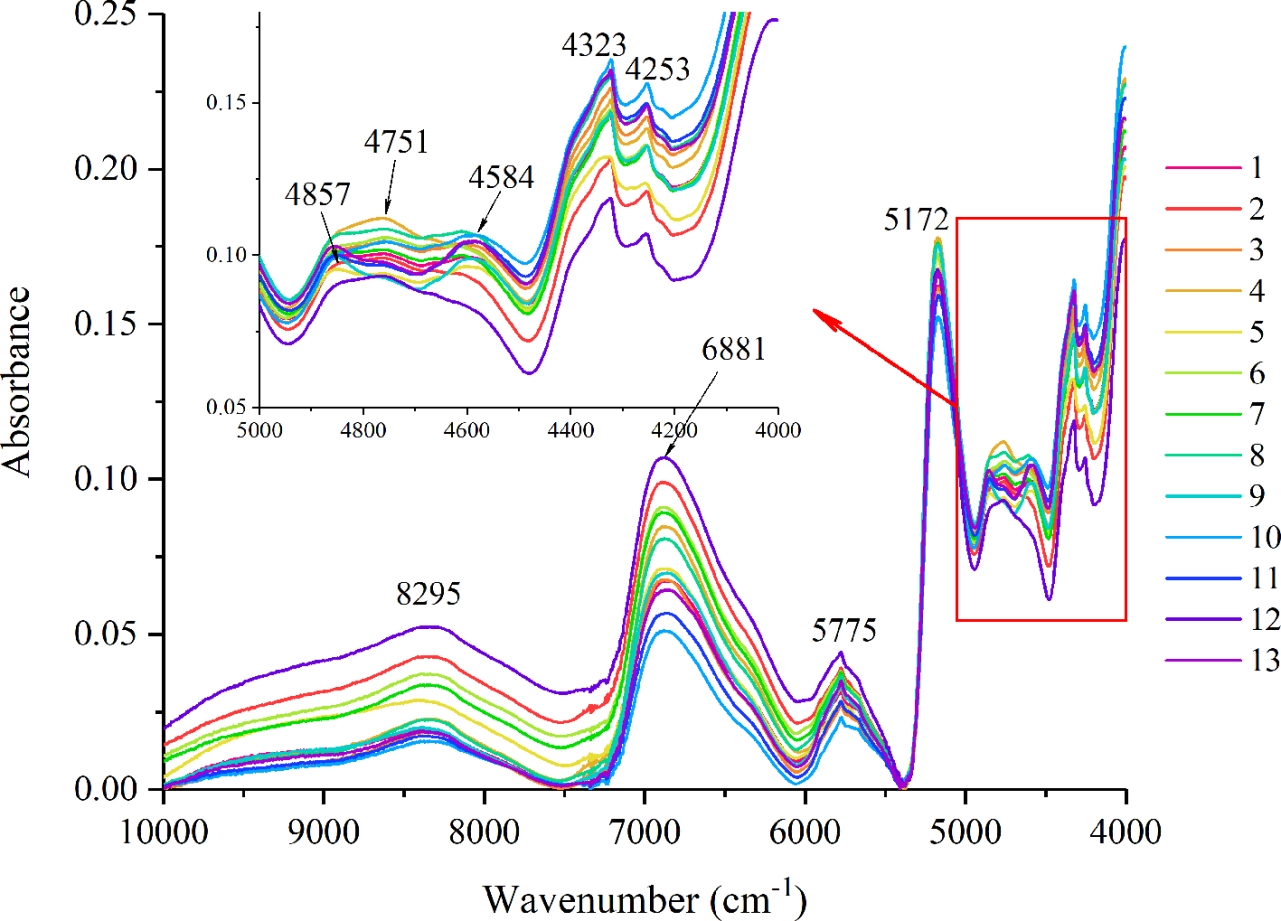
Stacked Fourier transform near-infrared (FT-NIR) spectra of *Eucommia ulmoides* leaves (EUL) from thirteen geographical regions.

For a better interpretation of the peak alignment, the averaged FT-NIR spectra of 13 collection sites were stacked in **Figure 1**, in which the amplifying band between 5,000 and 4,000 cm^-1^ was shown on the upper left corner. The wideband at 8,295 cm^-1^ is the second overtone of C–H stretching vibrations of the CH_3_ and CH_2_ groups. An obvious peak at 6,881 cm^-1^ was the first overtone with O–H stretching, whereas the weak absorbance at 5775 cm^-1^ was C–H stretching of R–OHCH_3_. The sharpest peat at 5,172 cm^-1^ is O–H stretching and OH deformation of H_2_O. The bands at 5,000–4,000 cm^-1^ were the C–H stretching and C–O group frequencies of carbohydrates and C–H stretching and deformation group frequencies of polysaccharide. Intuitively, an absorbance difference was observed among different geographical origins except for the band at 4,584 cm^-1^, which indicated the sample spectra from Guizhou and Anhui provinces. The whole peak explanation of NIR spectra is summarized in **Table 2**.

**Table 2:** Peak assignments on the FT-NIR and ATR-FT-MIR spectra of EUL.

Spectral type	Wavenumber (cm^-1^)	Assignments
NIR	8,295	Second overtone of C–H stretching vibrations of CH_3_ and CH_2_ groups
	6,881	First overtone with O-H stretching
	5,775	C–H stretching of R–OHCH_3_
	5,172	O–H stretching and OH deformation of H_2_O
	5,000–4,000	C–H stretching and C═O group frequencies of carbohydrates and C–H stretching and deformation group frequencies of polysaccharide
MIR	3,317	O–H stretching of polysaccharide and amide A of proteins
	2,919, 2,851	Asymmetric and symmetric C–H stretching of CH_2_
	1,734	C═O stretching of lipids, etc.
	1,629	Amide I band
	1,607	C═O stretching of flavones
	1,553	Amide II band
	1,439	C–H scissoring and in-plane deforming
	1,375	CH_3_ scissoring
	1,317	α-Helix of amide III band
	1,243	Amide III and C–O stretching
	1,145	C–O–C stretching
	1,101, 1,068, 1,054	Polysaccharide rings
	920	Sugar skeleton vibration

**Figure 2:**
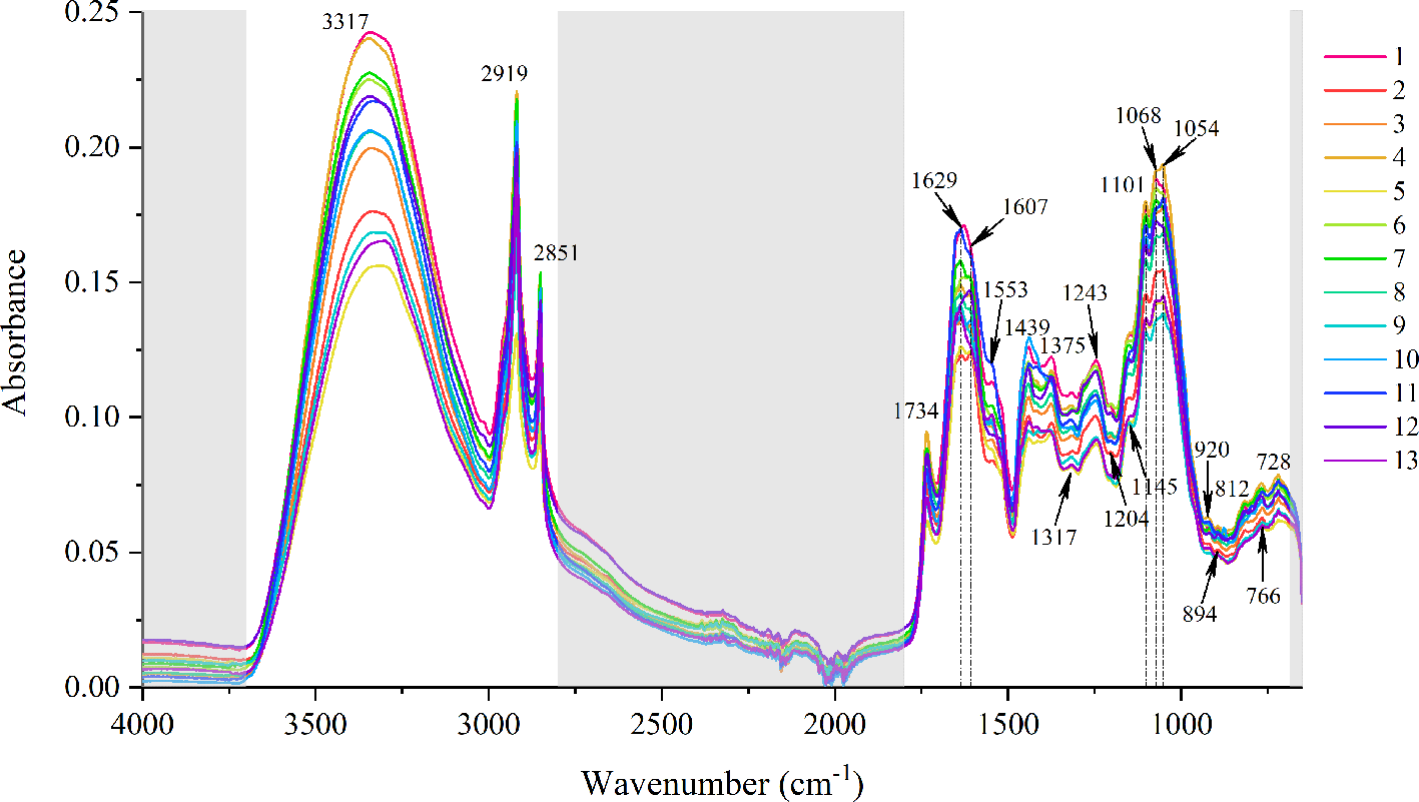
Stacked attenuated total reflection Fourier transform mid-infrared (ATR-FT-MIR) spectra of *Eucommia ulmoides* leaves (EUL) from 13 geographical regions.

As for the qualitative analysis of ATR-FT-MIR spectra, the averaged spectra of 13 geographical origins were also displayed in **Figure 2**. After excluding the non-related spectra variables with chemical information of the herb in the baseline area and the absorbance of diamond crystal and CO_2_, the spectra variables could reflect most of the chemical structure information including polysaccharide, amide, lipids and flavones. The detailed peak alignments are summarized in **Table 2**. For instance, the absorbance peak at 1,607 cm^-1^ is incorporated with the C═O stretching of flavones. A total of 36 flavonoids have been reported in *E. ulmoides* (Wang et al., 2019), including quercetin, astragalin, rutin, and hyperin, etc. At visual, there is no obvious difference among these averaged spectra from different geographical origins.

### Exploratory Analysis

In general, exploratory analysis is the initial step to investigate the original cluster results with part of variables or the whole dataset. In our present research, PCA was used as one of the methods used for the first two PCs to explain part of spectra information. t-SNE and HCA were utilized for an initial cluster with integrated variables including ATR-FT-MIR and FT-NIR spectra. The combination graphs comprised of score plots from two kinds of exploratory methods due to the fact that both of PCA and t-SNE displayed the cluster results in the form of a 2D score plot. The climatic zoning maps are shown below.

**Figure 3:**
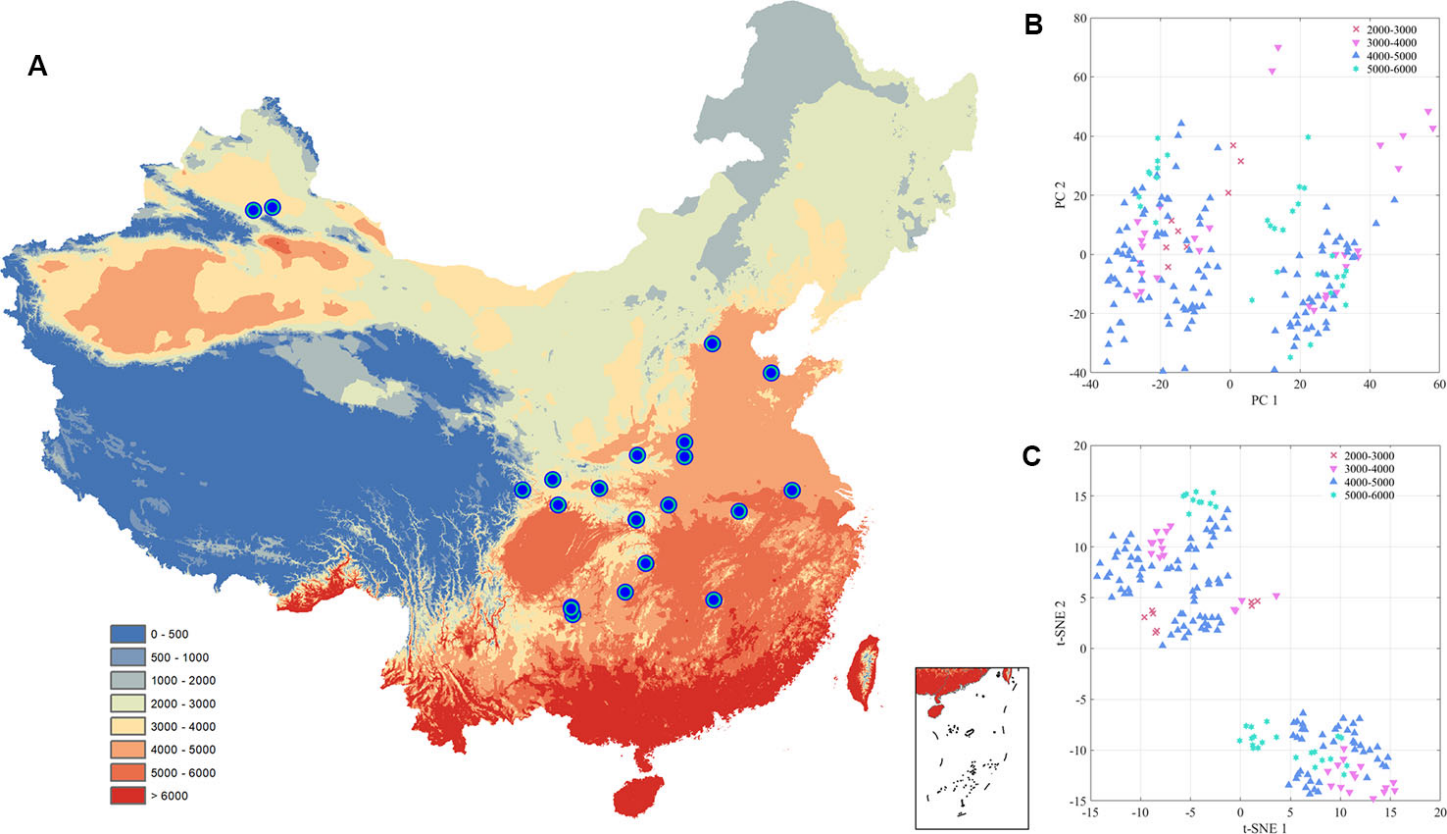
The exploratory analysis results of *Eucommia ulmoides* leaves (EUL) samples in ≥10°C accumulated temperature **(A)**: The distribution of each collection site; **(B)**: PCA; **(C)**: t-SNE.

**Figure 4:**
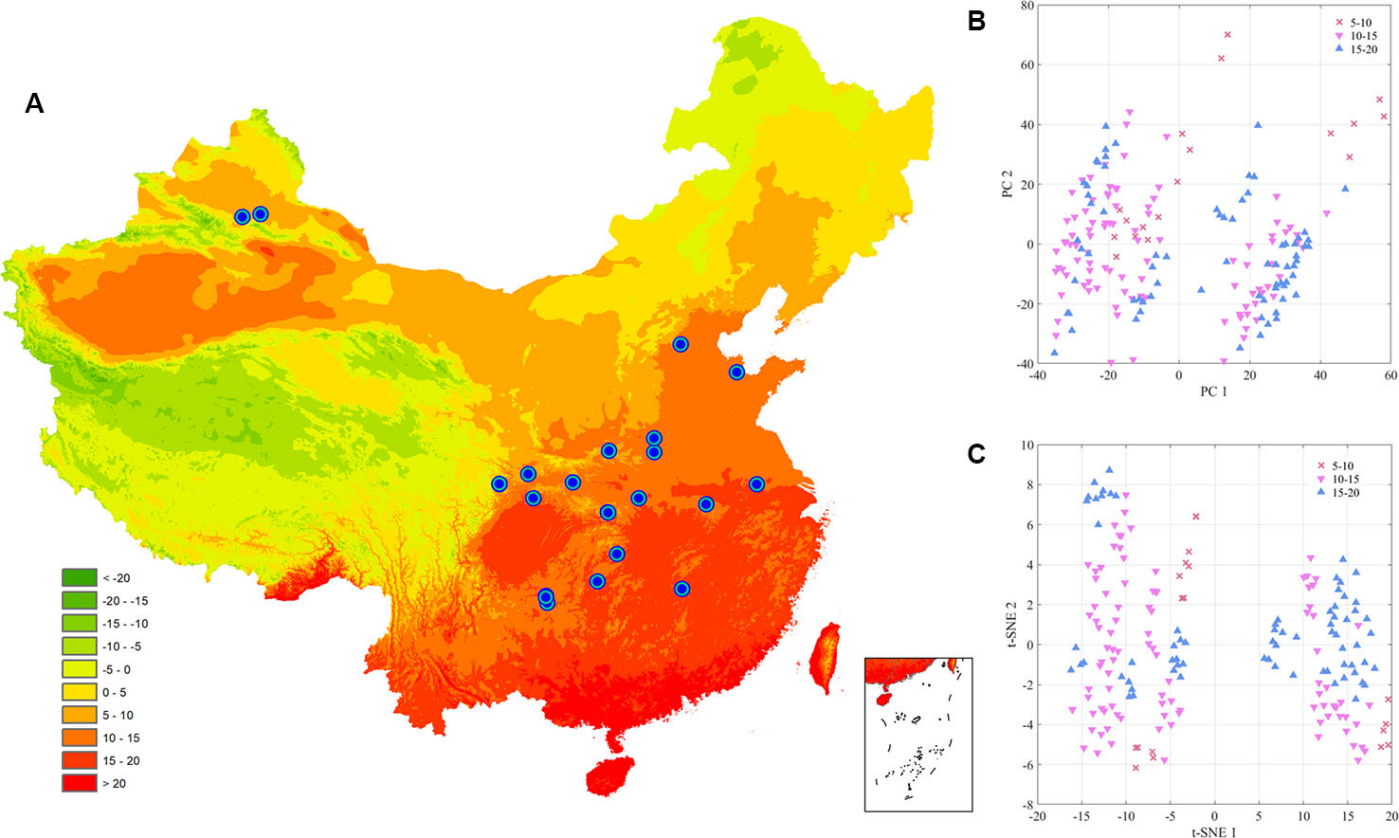
The exploratory analysis results of *Eucommia ulmoides* leaves (EUL) samples in annual average temperature **(A)**: The distribution of each collection site; **(B)**: PCA; **(C)**: t-SNE.

**Figure 5:**
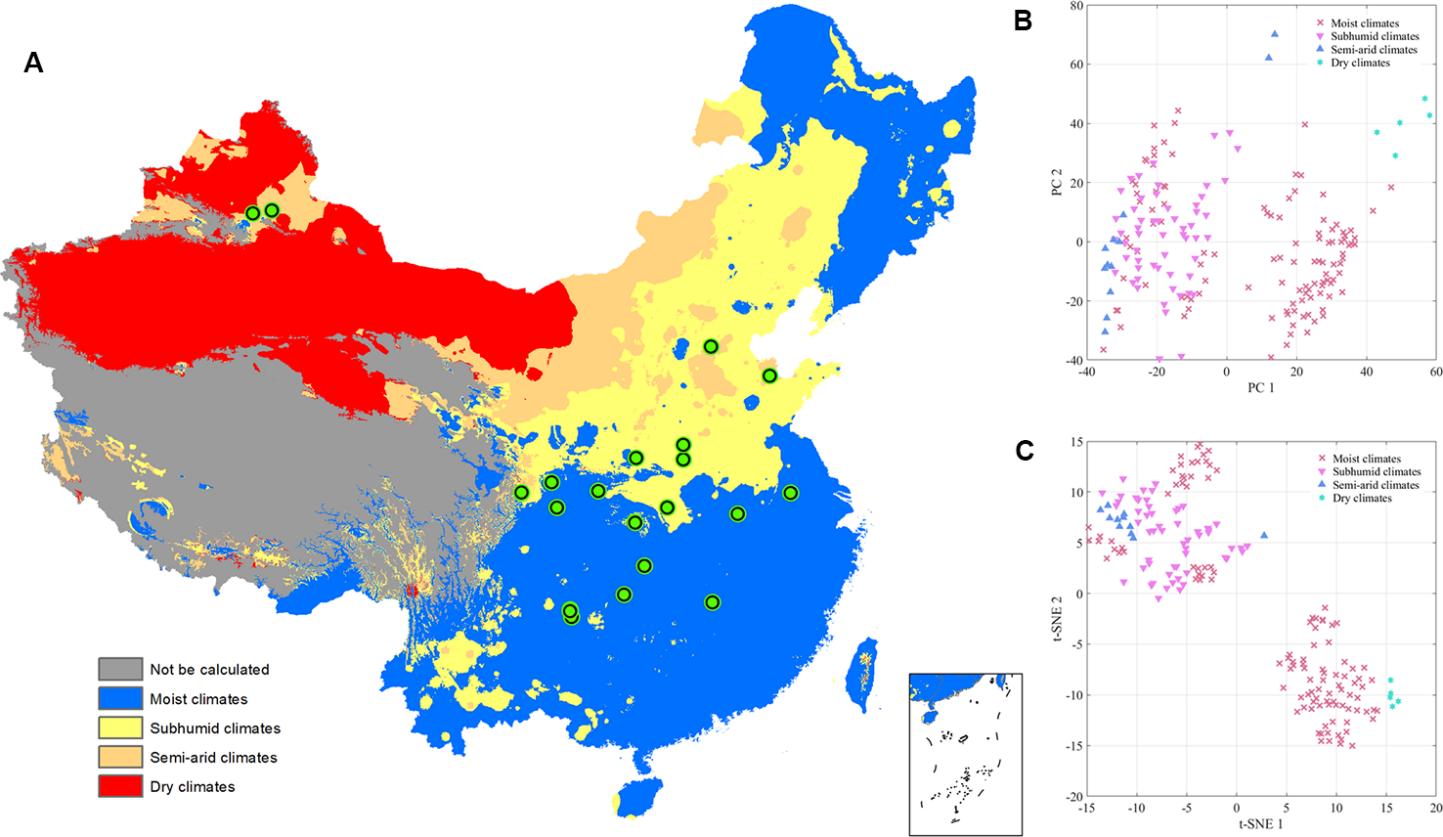
The exploratory analysis results of *Eucommia ulmoides* leaves (EUL) samples in dryness **(A)**: The distribution of each collection site; **(B)**: PCA; **(C)**: t-SNE.

**Figure 6:**
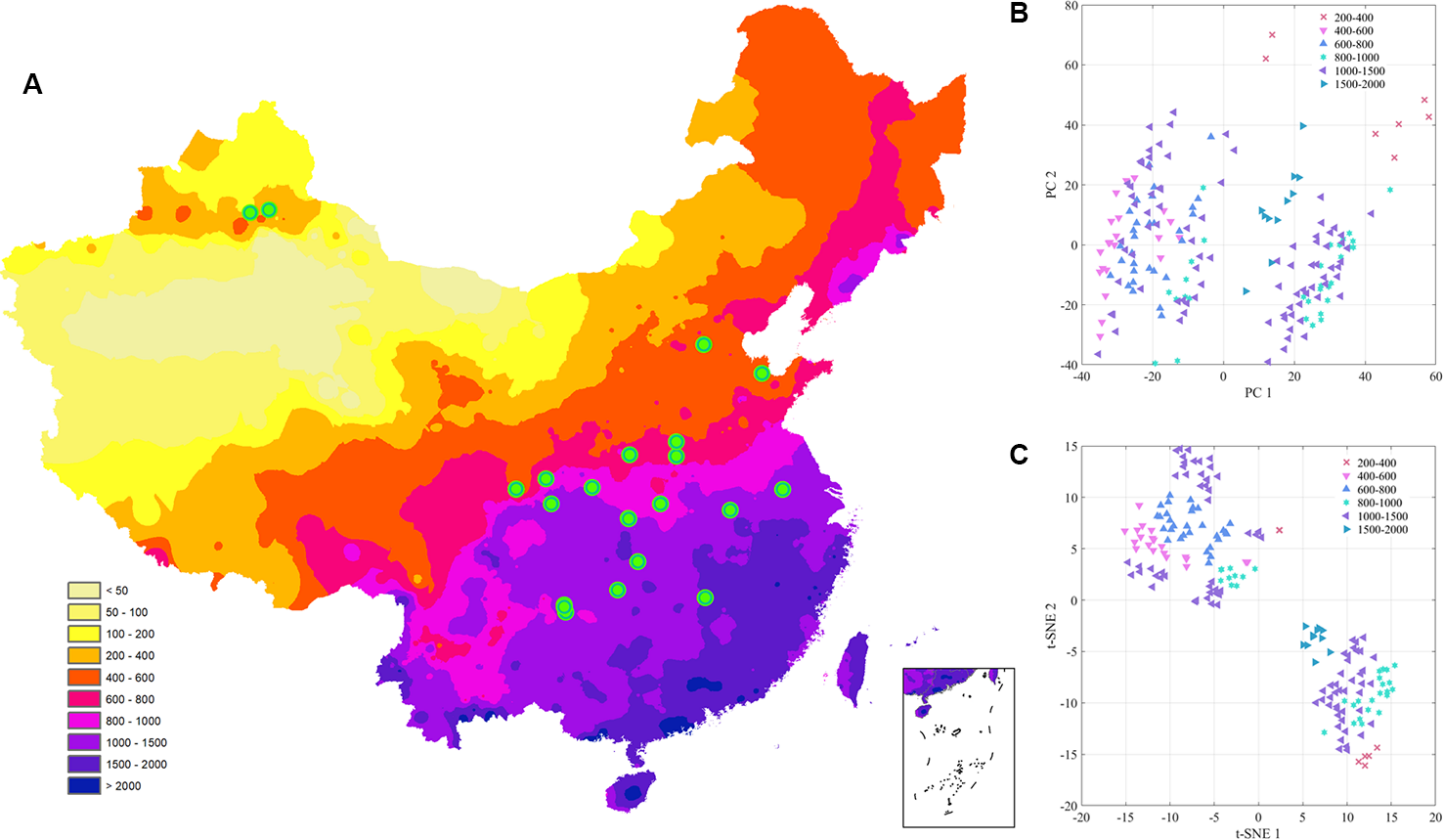
The exploratory analysis results of *Eucommia ulmoides* leaves (EUL) samples in annual average precipitation **(A)**: The distribution of each collection site; **(B)**: PCA; **(C)**: t-SNE.

**Figure 7:**
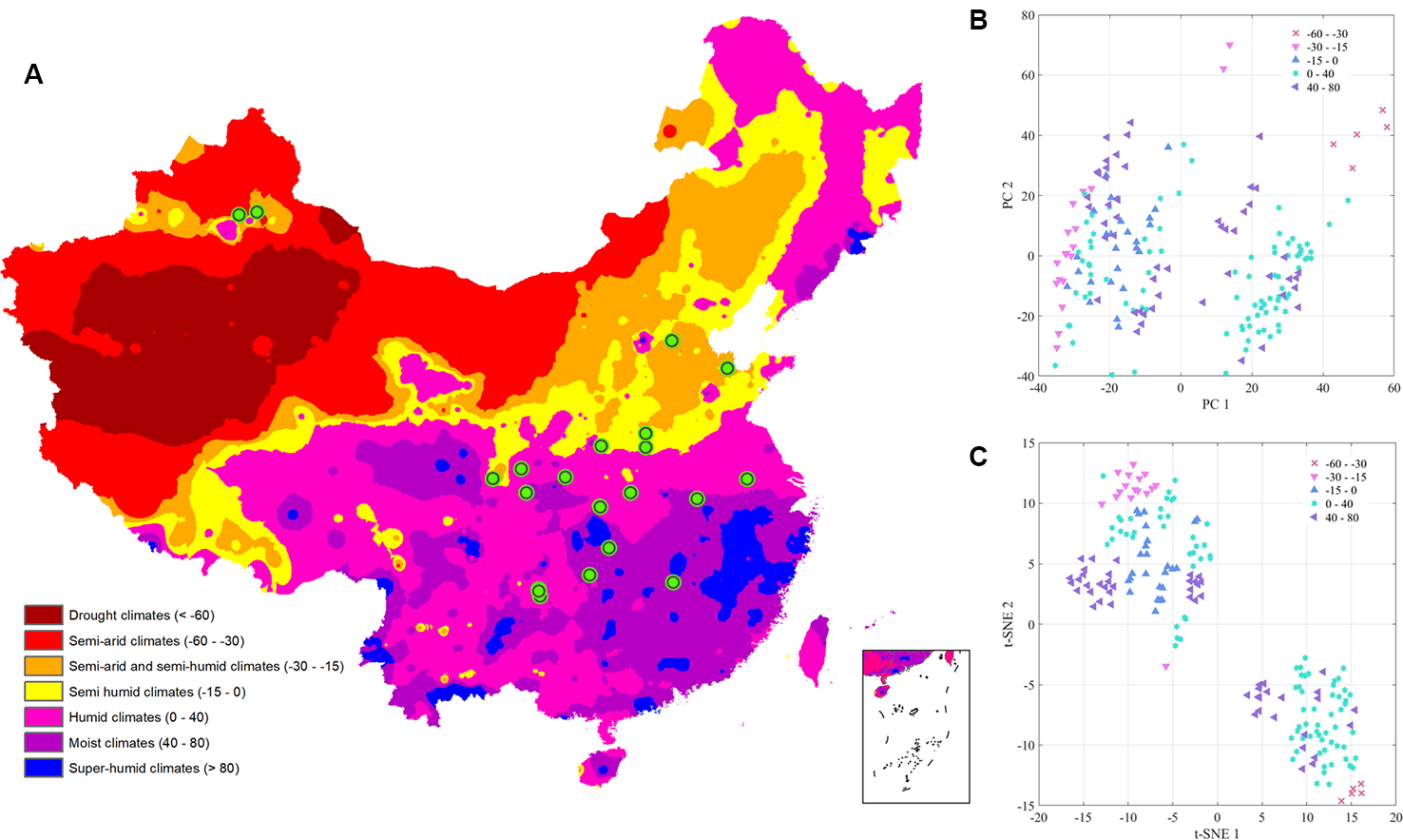
The exploratory analysis results of *Eucommia ulmoides* leaves (EUL) samples in moisture index **(A)**: The distribution of each collection site; **(B)**: PCA; **(C)**: t-SNE.

**Figure 8:**
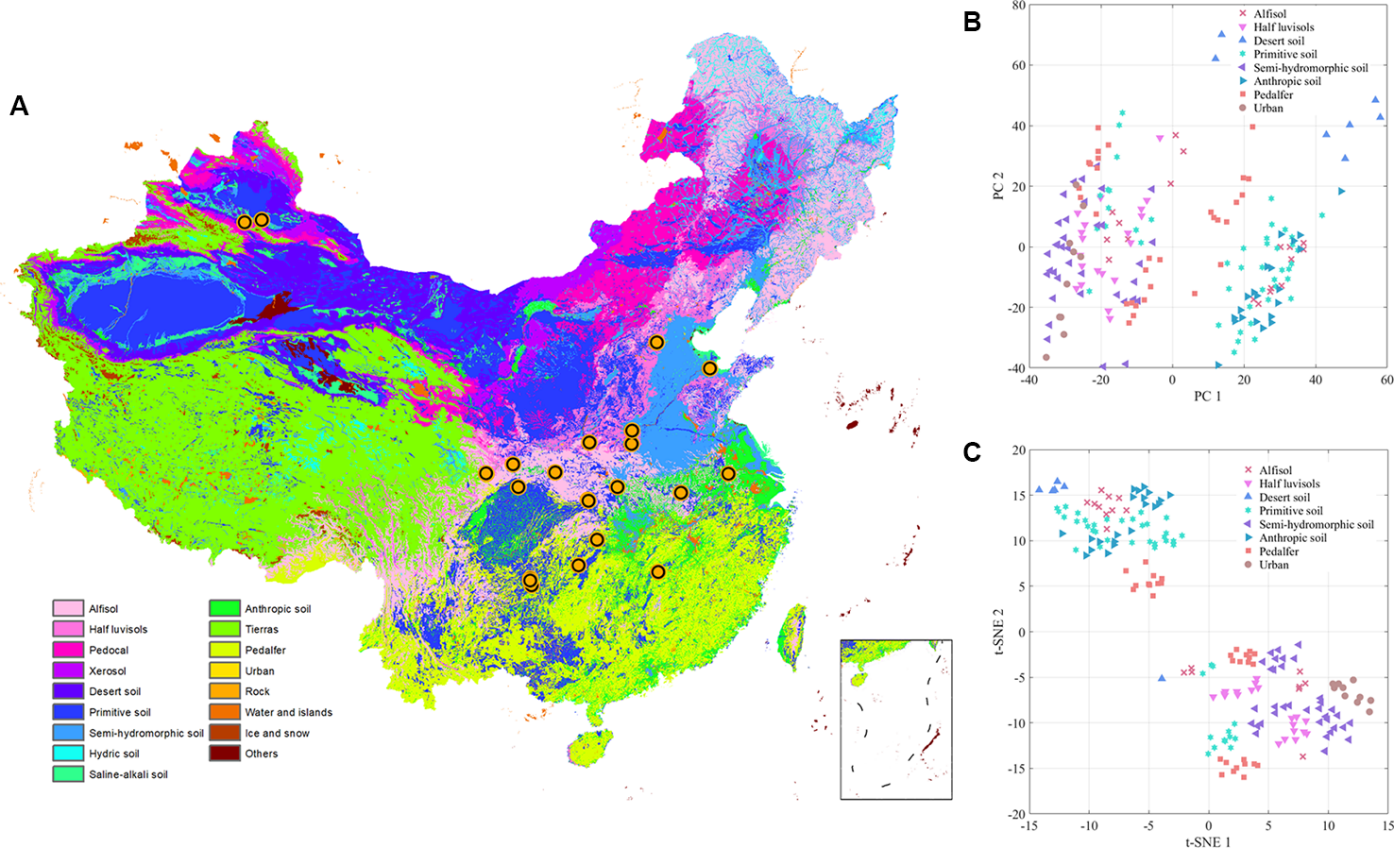
The exploratory analysis results of *Eucommia ulmoides* leaves (EUL) samples in soil type **(A)**: The distribution of each collection site; **(B)**: PCA; **(C)**: t-SNE.

Accumulated temperature (≥10C°) distribution in China and samples cluster results based on the climatic conditions are shown in **Figure 3**, which displays that there was no clear cluster performance with the variation of the climatic conditions. Similar to the classification tendency above, the exploratory results of annual average temperature (**Figure 4**) also did not show a clear cluster according to the climatic regionalization of temperature by either using PCA or t-SNE. Score plots of t-SNE were mentioned below because of the excellent visualization performance of t-SNE. Besides, three climatic factors (dryness, annual average precipitation, and moisture index) were used to investigate further cluster tendency. Their cluster results were shown in **Figures 5**–**7**, which reflect an interesting focus that five samples from Xinjiang Autonomous Region were distributed in an individual group, whereas two samples from the same location could not be distributed in the same cluster. Comparing two locations in the province, we found that the humidity index differed (**Table S1**) between two cities (Ürümqi city=-39.84, Fukang City=-19.79). As for the soil type that caused the cluster difference, there were samples from Nanjing, Jiangsu Province that showed individual classification in **Figure 8**, which indicated that these samples were grown in urban soil, which was different from other soil types. Generally, the cluster results of all of samples were sophisticated in terms of various climatic conditions. The reason why these samples could not be classified according to the individual classification of the same climatic condition is that the chemical information reflected by two of spectra is influenced by these climatic parameters simultaneously.

**Figure 9:**
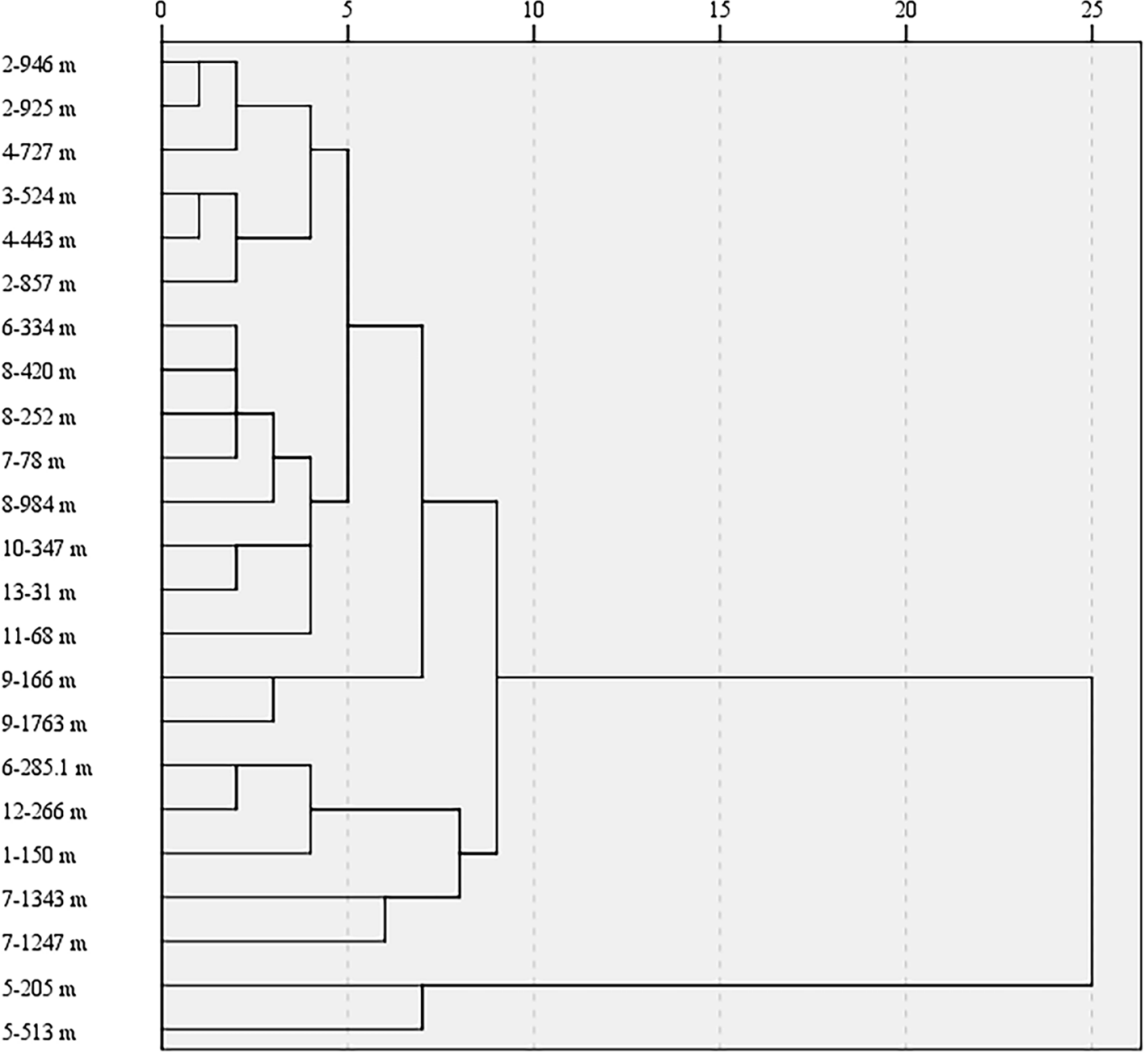
Hierarchical cluster analysis (HCA) dendrograms of the *Eucommia ulmoides* leaves (EUL) samples from different regions (The hyphen is preceded by the region of EUL samples, the hyphen followed by each sample collection site elevation).

To display more detailed visualization results, these samples from 23 collection sites were analyzed with HCA method, because each sample has precise classification with a direct measurement distance (**Figure 9**). When the distance was equal to 10, these samples were divided into two categories, in which samples from Xinjiang Autonomous Region were placed in a sole class, whereas other samples from other collection regions were clustered as one class. The interesting cluster could be interpreted that these leaves from Xinjiang have special cultivation mode. In the cultivation area, the aerial parts of these trees were cut in winter because of the extremely low temperature. Then, the young leaves from the young branch were collected. However, leaves from other locations were collected from plants whose main branch remained even in winter. In addition, these samples from Xinjiang were located at high-latitude regions in China, which might be the reason for the individual cluster. When the distance was equal to 9, these samples were divided into three classes. Those samples from Xinjiang still belonged to an individual category, whereas samples from Hubei (Shennongjia), Jiangxi, Anhui, and Hunan (Jishou city) were regarded as the second class. The remaining samples were divided as the third class. Samples from Xiangyang city in Hubei Province were different from samples obtained from two collection sites in the same province, which indicated that the altitude was the main influencing factor to the formation of two clusters. Moreover, chemical variation caused by altitude has been reported in published literatures. Li et al. (2016) have found that elevations of collection locations affect the chemical components of wild *Wolfporia extensa* sclerotia analyzed using mid-infrared spectra combined with the same HCA method. In addition, Sun et al. (2016) also indicated that 3,000 m was the boundary where the contents of nine constituents in the >3,000-meter group of medicinal rhubarb were significantly higher (*P*<0.05) compared with the <3,000-meter group.

### Results of PLS-DA

It has been mentioned in the chapter of theory explanation that three fusion strategies were used for discrimination of geographical origins of the herb. The calculation results in the form of a confusion matrix of low-level fusion in the calibration set are displayed in **Table S2**, and the discrimination of validation set is shown in **Table S3**. The results of low-level fusion indicated that all samples in the calibration set were accurately classified into their respective categories, whereas 83.61% samples in the validation set were discriminated accurately. Furthermore, mid-level fusion with combined principal components from two kinds of spectra was utilized to investigate the discrimination performance. The results (shown in **Tables S4** and **S5**) indicated that both of accuracy rates were lower than those of low-level fusion strategy (Accuracy of calibration set=87.30%, Accuracy of validation set=77.05%). The poor discrimination performance of the latter fusion method could be interpreted that fused principal components from two kinds of spectra failed to reflect difference among 13 geographical origins although there was little variable for short calculation time. Therefore, high-level fusion strategy was further applied to investigate model discrimination performance. Based on the fuzzy set theory, individual discrimination results from two spectroscopies that were recalculated for more precise classification. According to the confusion matrix of calibration (**Table S6**) and validation set (**Table S7**), 100% of samples in the calibration set were accurately discriminated with excellent model parameters (SEN, SPE, PRE, and EFF were equal to 100%), and this result was the same as the results of low-level fusion. However, 86.89% samples of the validation set in high-level fusion strategy were classified into their correct classes, and this percentage was higher than obtained by the low-level fusion method. Particularly, samples from Jiangxi, Xinjiang, Jiangsu, Anhui, and Shandong Provinces had four model parameters equal to 100%, and this result indicated that these sample show a more obvious difference in terms of chemical information reflecting by FT-NIR and ATR-FT-MIR spectra. The advantage of high-level fusion strategy has also been reported for the geographical traceability of *Paris polyphylla* var. *yunnanensis* (Wu et al., 2018) and *Panax notoginseng* (Li et al., 2018).

To validate the model robustness and fitting of PLS-DA, a permutation test was used with values of six parameters, namely, R^2^ minimum, R^2^ maximum, Q^2^ minimum, Q^2^ maximum, R^2^-intercept, and Q^2^-intercept. Evaluation results of low-level fusion in **Table 3** show that the calibration set had an excellent model performance with 100% model evaluation parameters and low error (RMSEE=0.05-0.13; RMSECV=0.14-0.30). As for the model parameters of the validation set, validation samples from Jiangsu, Shaanxi, Xinjiang, Jiangsu, and Anhui have 100% model evaluation parameters with RMSEP from 0.10 to 0.20. Permutation test of low-level fusion indicated that the model was robust and fitting in which R^2^ and Q^2^ were lower than the original R^2^ and Q^2^ whereas the Q^2^-intercept was lower than 0. Results of model evaluation parameters and permutation test in mid-level fusion approach are shown in **Table 4**. Compared with the model parameters of low-level fusion, mid-level fusion strategy had poorer model evaluation parameters had the lowest SEN, SPE, PRE, and EFF. Moreover, RMSEE and RMSECV in the calibration set were higher than those of low-level fusion, whereas the averaged RMSEP was also more than 0.02 of that of low-level fusion. Permutation test of mid-fusion strategy also indicated that there was an over-fitting risk in terms of the comparison between R^2^ and original R^2^ and between Q^2^ and original Q^2^.

**Table 3:** Classification parameters obtained for PLS-DA model using low-level fusion of EUL with different collection regions.

Parameters	1	2	3	4	5	6	7	8	9	10	11	12	13	Average
Calibration set														
SEN (%)	100.00	100.00	100.00	100.00	100.00	100.00	100.00	100.00	100.00	100.00	100.00	100.00	100.00	100.00
SPE (%)	100.00	100.00	100.00	100.00	100.00	100.00	100.00	100.00	100.00	100.00	100.00	100.00	100.00	100.00
PRE (%)	100.00	100.00	100.00	100.00	100.00	100.00	100.00	100.00	100.00	100.00	100.00	100.00	100.00	100.00
EFF (%)	100.00	100.00	100.00	100.00	100.00	100.00	100.00	100.00	100.00	100.00	100.00	100.00	100.00	100.00
RMSEE	0.08	0.13	0.09	0.10	0.05	0.11	0.10	0.12	0.06	0.07	0.07	0.09	0.08	0.09
RMSECV	0.15	0.28	0.20	0.24	0.19	0.23	0.23	0.30	0.17	0.14	0.17	0.18	0.18	0.20
Validation set														
SEN (%)	100.00	100.00	33.33	100.00	100.00	57.14	100.00	100.00	0	100.00	0	100.00	66.67	73.63
SPE (%)	100.00	96.08	100.00	100.00	100.00	100.00	96.43	88.24	100.00	100.00	100.00	100.00	100.00	98.52
PRE (%)	100.00	83.33	100.00	100.00	100.00	100.00	71.43	62.50	–	100.00	–	100.00	100.00	92.48
EFF (%)	100.00	98.02	57.74	100.00	100.00	75.59	98.20	93.93	0	100.00	0	100.00	81.65	77.32
RMSEP	0.15	0.26	0.17	0.20	0.10	0.27	0.18	0.28	0.15	0.13	0.15	0.15	0.17	0.18
Permutation test														
R^2^ (min–max)	0.66–0.84	0.66–0.87	0.55–0.85	0.73–0.85	0.26–0.88	0.66–0.83	0.67–0.85	0.69–0.86	0.47–0.84	0.72–0.83	0.65–0.86	0.59–0.83	0.65–0.85	–
Q^2^ (min–max)	-1.08–-0.08	-1.38–-0.33	-0.98–-0.13	-0.97–-0.15	-0.91–-0.01	-1.48–-0.37	-1.27–-0.30	-1.25–-0.05	-0.83–-0.21	-0.94–-0.21	-0.91–-0.18	-1.28–-0.26	-1.17–-0.27	–
Original R^2^	0.91	0.89	0.88	0.92	0.94	0.88	0.88	0.90	0.86	0.91	0.88	0.86	0.89	0.89
Original Q^2^	0.64	0.44	0.30	0.48	0.36	0.58	0.38	0.42	-0.22	0.70	0.21	0.48	0.47	0.40
Q^2^-intercept	-0.69	-0.80	-0.64	-0.60	-0.57	-0.85	-0.68	-0.71	-0.55	-0.70	-0.62	-0.78	-0.67	-0.68

**Table 4:** Classification parameters obtained for PLS-DA model using mid-level fusion of EUL with different collection regions.

Parameters	1	2	3	4	5	6	7	8	9	10	11	12	13	Average
Calibration set														
SEN (%)	100.00	100.00	0	85.71	100.00	92.31	100.00	100.00	0	100.00	75.00	71.43	100.00	78.80
SPE (%)	100.00	91.43	100.00	100.00	99.17	100.00	97.39	97.17	100.00	100.00	100.00	100.00	100.00	98.86
PRE (%)	100.00	70.00	–	100.00	83.33	100.00	78.57	86.96	–	100.00	100.00	100.00	100.00	92.62
EFF (%)	100.00	95.62	0	92.58	99.59	96.08	98.69	98.57	0	100.00	86.60	84.52	100.00	80.94
RMSEE	0.15	0.27	0.21	0.24	0.07	0.16	0.18	0.27	0.15	0.10	0.14	0.17	0.17	0.18
RMSECV	0.21	0.33	0.21	0.29	0.19	0.24	0.24	0.34	0.16	0.16	0.18	0.22	0.20	0.23
Validation set														
SEN (%)	75.00	100.00	0	100.00	100.00	57.14	100.00	100.00	0	100.00	0.00	33.33	66.67	64.01
SPE (%)	100.00	92.16	100.00	100.00	100.00	100.00	92.86	90.20	100.00	98.28	100.00	100.00	100.00	97.96
PRE (%)	100.00	71.43	–	100.00	100.00	100.00	55.56	66.67	–	75.00	–	100.00	100.00	86.87
EFF (%)	86.60	96.00	0	100.00	100.00	75.59	96.36	94.97	0	99.13	0.00	57.74	81.65	68.31
RMSEP	0.18	0.28	0.21	0.23	0.12	0.28	0.21	0.28	0.16	0.13	0.15	0.17	0.18	0.20
Permutation test														
R2 (min–max)	0.06–0.50	0.12–0.46	0.10–0.63	0.10–0.57	0.11–0.50	0.19–0.48	0.13–0.46	0.24–0.49	0.09–0.53	0.11–0.57	0.09–0.65	0.13–0.49	0.08–0.51	–
Q2 (min–max)	-0.69–-0.15	-0.79–-0.15	-0.78–-0.03	-0.81–-0.19	-0.67–-0.14	-1.09–-0.19	-0.62–-0.05	-0.88–-0.27	-0.58–-0.01	-0.77–-0.09	-0.72–-0.05	-0.64–-0.14	-0.64–-0.03	–
Original R^2^	0.59	0.50	0.23	0.48	0.87	0.75	0.63	0.50	0.14	0.83	0.42	0.49	0.48	0.53
Original Q^2^	0.21	0.25	0.11	0.12	0.49	0.41	0.32	0.20	-0.06	0.58	-0.05	0.13	0.25	0.23
Q2-intercept	-0.41	-0.50	-0.46	-0.44	-0.37	-0.55	-0.47	-0.56	-0.27	-0.45	-0.31	-0.39	-0.38	-0.43

### Results of RF

**Figure 10:**
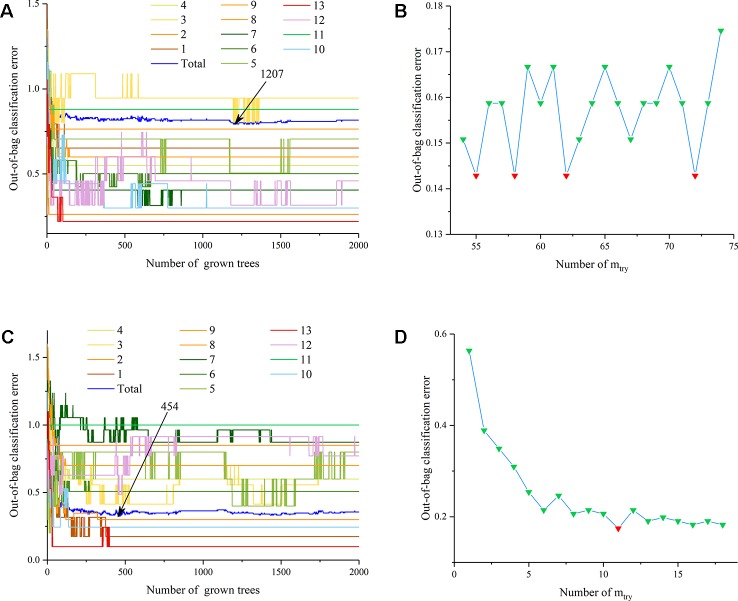
The parameter optimization of random forest models **(A)**: n_tree_ of the low-level data fusion dataset; **(B)**: m_try_ of the low-level data fusion dataset; **(C)**: n_tree_ of the mid-level data fusion dataset; **(D)**: m_try_ of the mid-level data fusion dataset).

Similar to the analysis of PLS-DA with three fusion methods, RF was also utilized for the discrimination analysis of the geographical origins of EULs. After selecting the best number of trees (n_tree_=1,207 in **Figure 10A**) and branch nodes (m_try_=55 in **Figure 10B**) with the lowest OOB error, results of low-level fusion in calibration set are displayed in **Table S8**. This table showed that the model distinguished samples from Jiangxi, Guizhou and Jiangsu effectively with 85.71% accuracy rate and 100% SEN, SPE, PRE, and EFF values. By contrast, the model could not classify these samples from other provinces in China because of the poor SEN and SPE values, and two parameters were used for interpretation of true positive and true negative rates, respectively. Results of low-level fusion in the validation set are displayed in **Table S9**, in which samples from Jiangxi, Shaanxi, Gansu, Jiangsu, and Anhui Provinces were accurately predicted as the respective geographical origins. As for other classes, model performance was the same as that of the calibration set. The model calibration of RF (85.71% accuracy rate) was poorer than that of PLS-DA (100% accuracy rate) with the same fusion strategy.

After the combination of principal components from two kinds of spectra, the establishment of the RF model was still needed to select the model parameters, including the best n_tree_ and the optimal branch nodes. Considering the OOB error in **Figures 10C, D**, the best number of trees is 454, and the m_try_ is 11, forming the RF model for the discrimination analysis of geographical origins. The detailed classification results of the calibration set with 81.75% accuracy rate are displayed in **Table S10**, which shows that Jiangsu and Jiangxi Provinces were highly and effectively discriminated as the correct origins of the corresponding samples. The detailed discrimination results of the validation set with 88.52% correct rate are shown in **Table S11**. The prediction results of the validation set indicated that the origin of samples from Jiangxi, Xinjiang, Jiangsu, Hebei, Anhui, and Shandong Provinces was predicted effectively and accurately.

**Figure 11:**
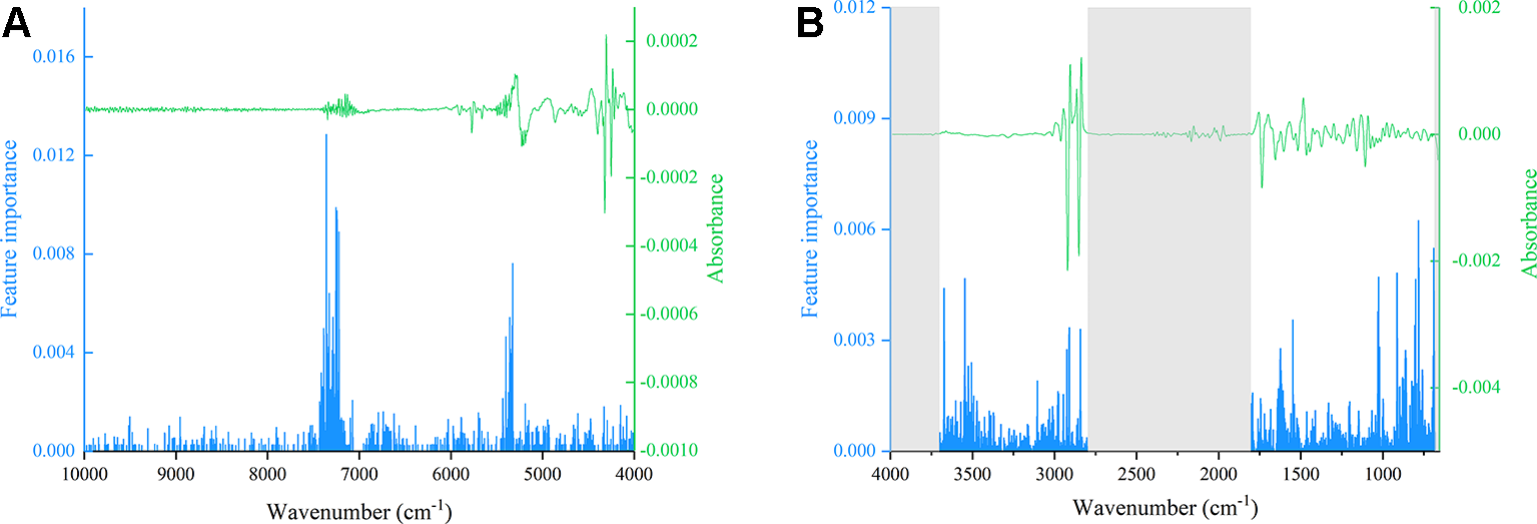
The permutation accuracy importance of each variable of spectra **(A)**: MSC+SD FT-NIR; **(B)**: MSC+SD ATR-FT-MIR).

Before the calculation of high-level fusion, individual spectra type was used for the model establishment to obtain vote value. **Figure S1** shows the initial selection results of two vital parameters, in which n_tree_ is 320 (**Figure S1A**) and m_try_ is 55 (**Figure S1C**) of FT-NIR spectra, whereas n_tree_ is 304 (**Figure S1B**) and m_try_ is 32 (**Figure S1D**) of ATR-FT-MIR. Based on the initial parameter results, 10-fold cross validation was used for selection of important variable in two kinds of spectra because these variables’ importance differed among different spectra bands. **Figure 11A** shows the feature importance of FT-NIR spectra, whereas **Figure 11B** shows the feature importance of ATR-FT-MIR. **Figure S2A** shows that 183 important variables in NIR spectra, and **Figure S2B** indicates 87 important variables in ATR-FT-MIR that should be used for further parameter selection. Finally, 1,311 trees (**Figure S2C**) and 61 branches (**Figure S2E**) were used as the model parameters in an FT-NIR model, whereas 365 trees (**Figure S2D**) and 9 branches (**Figure S2F**) were used as ATR-FT-MIR model parameters. After obtaining the vote results of RF from individual spectra, two votes of one sample from two spectra were weighted again, thereby forming the final results of the high-level fusion method. The detailed results of the calibration set were displayed in **Table S12** with 92.86% accuracy rate, whereas the confusion matrix of validation set was shown in **Table S13** with 93.44% correct rate. Four model evaluation parameters indicated that in calibration and validation sets, the model had high SEN and SPE, and RF with high-level fusion is precious and efficient.

In general, high-level fusion strategy combined with RF model with a high accuracy rate in the validation set was regarded as the best discrimination model for the determination of traceability of geographical origins in EUL quality control. However, the correct rate of calibration set was lower than that of PLS-DA with the same fusion method because of the over-fitting risk.

## Conclusion

Based on the non-destructive, fast and efficient advantages of FT-NIR and ATR-FT-MIR technologies, this work utilized chemometrics to identify EUL from different regions. RF is supplemented with high-level data fusion strategies compared with the traditional PLS-DA model. Geographical origins of EUL can be effectively distinguished from each production region. This may be due to the fact that high-level fusion occurs at the decision level and is less affected by irrelevant or interference information. The same as the PLS-DA model, the accuracy of low-level and mid-level fusion is relatively lower than that of high-level fusion.

The influence investigation of climate and soil type influences indicated that the cluster tendencies of all of the samples were sophisticated. These samples could not be classified according to the individual classification under the same climatic conditions, because the chemical information reflected by two of spectra was influenced by these climatic parameters simultaneously.

In general, our results were by no means exhaustive, but these findings can provide scientific support for EULs’ geographic authentication and can reveal chemical composition accumulation and changes in different environments. Moreover, different environmental factors that affect the cumulative changes of EUL chemical composition in different regions were revealed.

## Data Availability Statement

All datasets generated for this study are included in the article/**Supplementary Material**.

## Author Contributions

JL and Y-ZW conceived and designed the study. C-YW processed the experiment, data analysis and wrote the paper. LT and QZ processed plant collection and data collection and arrangement. Y-JL and LL finished the statistical analysis and manuscript revision.

## Funding

This work was supported by the National Natural Science Foundation of China (Grant No. 31960323), Special Funds Project for Central Government Guides Local Science and Technology Development (Grant No. 2018CT5012), National & Local United Engineering Laboratory of Integrative Utilization Technology of *Eucommia Ulmoides* Foundation (Grant No. NLE201701), Hunan Provincial Innovation Foundation for Postgraduate (Grant No. CX2018B725) and Natural Science Research Project of Jishou University (Grant No. Jdy1856 and Grant No. Jdx17020).

## Conflict of Interest

The authors declare that the research was conducted in the absence of any commercial or financial relationships.
