# Supplementary material for: Geographic Authentication of Eucommia ulmoides Leaves Using Multivariate Analysis and Preliminary Study on the Compositional Response to Environment
Source: Front Plant Sci. 2020 Feb 19;11:79. doi: 10.3389/fpls.2020.00079 (PMC7042207; doi:10.3389/fpls.2020.00079)
Supplement: Supplementary file 1 [file DataSheet_1.docx]

Supplementary Material

# Supplementary Figures and Tables

## Supplementary Figures


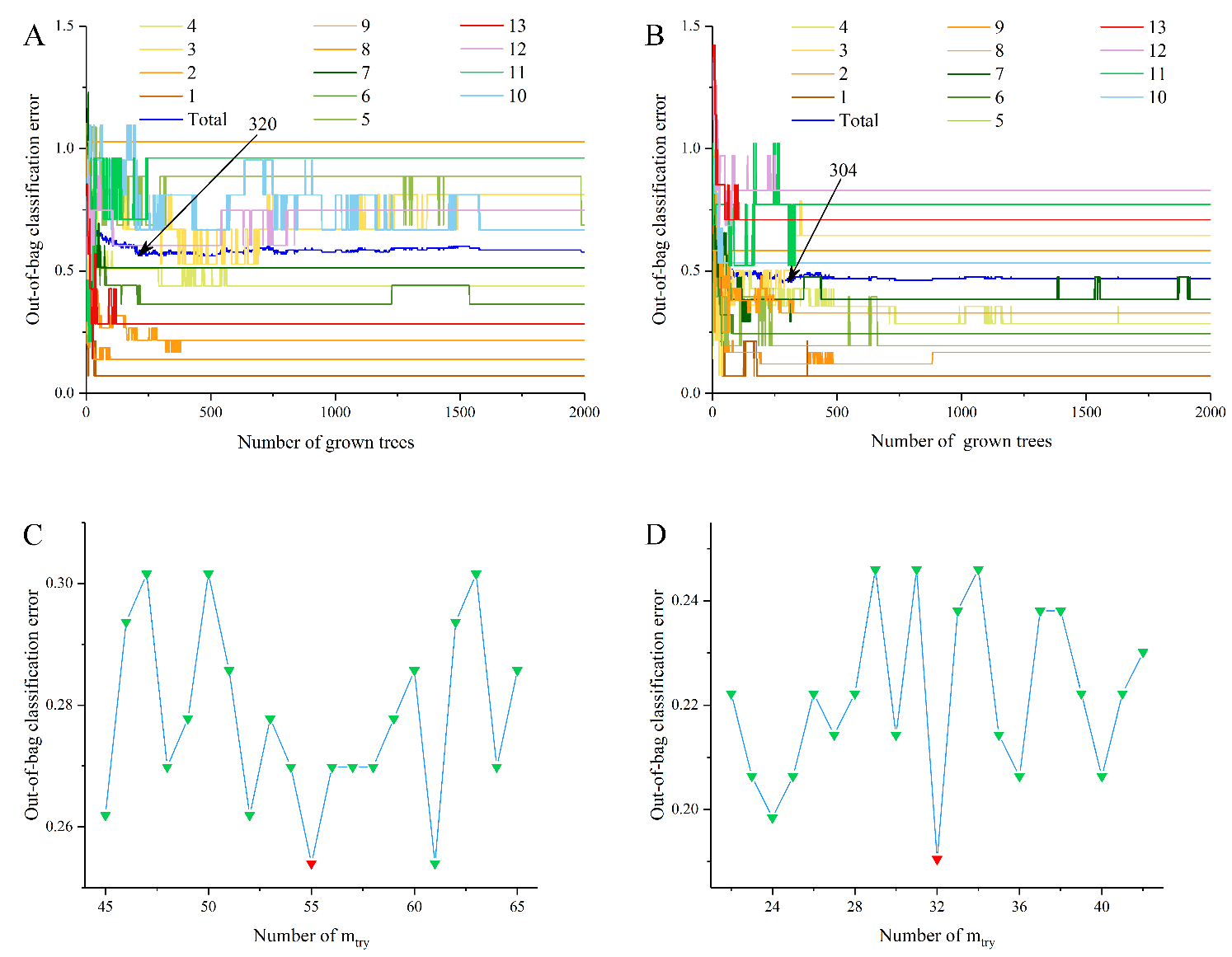


**Supplementary Figure 1.** The parameter optimization of random forest models using high-level data fusion without selection of important variables (A: n_tree_ of the FT-NIR dataset; B: m_try_ of the ATR-FT-MIR dataset; C: n_tree_ of the FT-NIR dataset; D: m_try_ of the ATR-FT-MIR dataset).


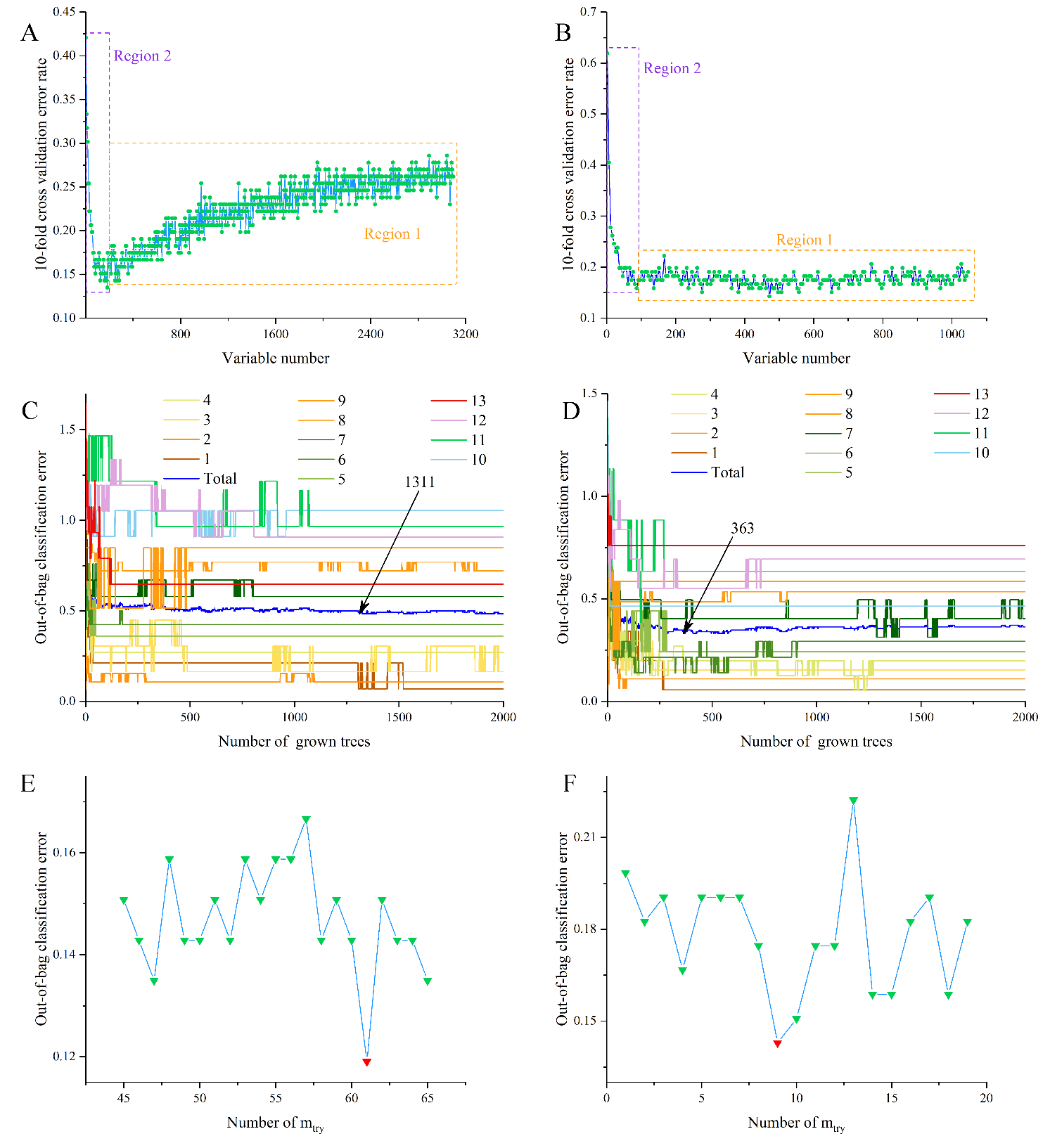


**Supplementary Figure 2.** The parameter optimization of random forest models using high-level data fusion with selection of important variables (A: Important variables selection of FT-NIR; B: Important variables selection of ATR-FT-MIR; C: n_tree_ of the FT-NIR dataset; D: m_try_ of the ATR-FT-MIR dataset; E: n_tree_ of the FT-NIR dataset; F: m_try_ of the ATR-FT-MIR dataset).

## Supplementary Tables

Table S1 Climate information and soil type of the EUL samples.

| Collection site | climatic regionalization | ≥10 °C accumulated temperature | Dryness | | annual average precipitation | Annual average temperature | Moisture index | Soil type |
| --- | --- | --- | --- | --- | --- | --- | --- | --- |
|  |  |  | Value | Category |  |  |  |  |
| Pingxiang City, Jiangxi Province | Middle subtropics | 5601.7 | 0.678 | Moist climates | 1580.9 | 16.8 | 74.04 | Pedalfer |
| Zunyi City, Guizhou Province | Middle subtropics | 4570.5 | 0.807 | Moist climates | 1046.1 | 14.4 | 34.16 | Primitive soil |
| Zunyi City, Guizhou Province | Middle subtropics | 4545.7 | 0.813 | Moist climates | 1080.9 | 14.4 | 34.47 | Anthropic soil |
| Zunyi City, Guizhou Province | Middle subtropics | 4548.9 | 0.811 | Moist climates | 1075.2 | 14.4 | 34.46 | Primitive soil |
| Guangyuan city, Sichuan Province | Middle subtropics | 5262.5 | 0.628 | Moist climates | 1359.6 | 15.9 | 60.61 | Primitive soil |
| Ankang City, Shaanxi Province | Northern subtropics | 4347.1 | 0.812 | Moist climates | 991.3 | 14.3 | 22.4 | Anthropic soil |
| Hanzhong City, Shaanxi Province | Northern subtropics | 3754 | 0.776 | Moist climates | 881.9 | 11.8 | 23.65 | Alfisol |
| Ürümqi City, Xinjiang Autonomous Region | Middle temperate zone | 3656 | 4.202 | Dry climates | 209.8 | 7.1 | -39.84 | Desert soil |
| Fukang City, Xinjiang Autonomous Region | Middle temperate zone | 3108.3 | 2.587 | Semi-arid climates | 322.7 | 6.9 | -19.79 | Desert soil |
| Zhangjiajie City, Hunan Province | Middle subtropics | 4952.9 | 0.723 | Moist climates | 1376 | 15.5 | 57.47 | Pedalfer |
| Jishou City, Hunan Province | Middle subtropics | 5038.1 | 0.75 | Moist climates | 1343.4 | 16 | 54.09 | Pedalfer |
| Shennongjia Forestry District, Hubei Province | Northern subtropics | 2630.6 | 1.082 | Subhumid climates | 1034 | 9.2 | 15.22 | Alfisol |
| Xiangyang city, Hubei province | Northern subtropics | 4567.6 | 1.146 | Subhumid climates | 858.1 | 14.8 | 5.76 | Semi-hydromorphic soil |
| Shennongjia Forestry District, Hubei Province | Northern subtropics | 3292.7 | 1.085 | Subhumid climates | 1035.5 | 9.5 | 15.2 | Primitive soil |
| Pingdingshan City, Henan Province | South temperate zone | 4450.8 | 1.169 | Subhumid climates | 736.9 | 13.8 | -2.84 | Half luvisols |
| Lingbao City, Henan Province | South temperate zone | 3704.8 | 1.067 | Subhumid climates | 677.8 | 10.3 | 3.38 | Half luvisols |
| Xingyang City, Henan Province | South temperate zone | 4511.7 | 1.411 | Subhumid climates | 603.9 | 13.8 | -14.39 | Semi-hydromorphic soil |
| Longnan City, Gansu Province | Northern subtropics | 2431.7 | 1.478 | Subhumid climates | 567.6 | 8.7 | -12.1 | Alfisol |
| Longnan City, Gansu Province | Northern subtropics | 2434.7 | 1.474 | Subhumid climates | 570.4 | 8.7 | -11.48 | Alfisol |
| Nanjing City, Jiangsu Province | Northern subtropics | 4978.5 | 0.892 | Moist climates | 1071.7 | 15.2 | 27.15 | Urban |
| Dingzhou City, Hebei Province | South temperate zone | 4407.8 | 1.382 | Subhumid climates | 544.3 | 12.3 | -17.76 | Semi-hydromorphic soil |
| Lu'an City, Anhui Province | Northern subtropics | 4167.6 | 0.762 | Moist climates | 1309.8 | 12.9 | 55.32 | Primitive soil |
| Linzi City, Shandong Province | South temperate zone | 4420.8 | 1.512 | Semi-arid climates | 519.6 | 12.6 | -19.58 | Semi-hydromorphic soil |

Table S2 Confusion matrix of PLS-DA in calibration set using low-level fusion.

| Class | 1 | 2 | 3 | 4 | 5 | 6 | 7 | 8 | 9 | 10 | 11 | 12 | 13 |
| --- | --- | --- | --- | --- | --- | --- | --- | --- | --- | --- | --- | --- | --- |
| 1 | 7 | 0 | 0 | 0 | 0 | 0 | 0 | 0 | 0 | 0 | 0 | 0 | 0 |
| 2 | 0 | 21 | 0 | 0 | 0 | 0 | 0 | 0 | 0 | 0 | 0 | 0 | 0 |
| 3 | 0 | 0 | 7 | 0 | 0 | 0 | 0 | 0 | 0 | 0 | 0 | 0 | 0 |
| 4 | 0 | 0 | 0 | 14 | 0 | 0 | 0 | 0 | 0 | 0 | 0 | 0 | 0 |
| 5 | 0 | 0 | 0 | 0 | 5 | 0 | 0 | 0 | 0 | 0 | 0 | 0 | 0 |
| 6 | 0 | 0 | 0 | 0 | 0 | 13 | 0 | 0 | 0 | 0 | 0 | 0 | 0 |
| 7 | 0 | 0 | 0 | 0 | 0 | 0 | 11 | 0 | 0 | 0 | 0 | 0 | 0 |
| 8 | 0 | 0 | 0 | 0 | 0 | 0 | 0 | 20 | 0 | 0 | 0 | 0 | 0 |
| 9 | 0 | 0 | 0 | 0 | 0 | 0 | 0 | 0 | 3 | 0 | 0 | 0 | 0 |
| 10 | 0 | 0 | 0 | 0 | 0 | 0 | 0 | 0 | 0 | 7 | 0 | 0 | 0 |
| 11 | 0 | 0 | 0 | 0 | 0 | 0 | 0 | 0 | 0 | 0 | 4 | 0 | 0 |
| 12 | 0 | 0 | 0 | 0 | 0 | 0 | 0 | 0 | 0 | 0 | 0 | 7 | 0 |
| 13 | 0 | 0 | 0 | 0 | 0 | 0 | 0 | 0 | 0 | 0 | 0 | 0 | 7 |

Table S3 Confusion matrix of PLS-DA in validation set using low-level fusion.

| Class | 1 | 2 | 3 | 4 | 5 | 6 | 7 | 8 | 9 | 10 | 11 | 12 | 13 |
| --- | --- | --- | --- | --- | --- | --- | --- | --- | --- | --- | --- | --- | --- |
| 1 | 4 | 0 | 0 | 0 | 0 | 0 | 0 | 0 | 0 | 0 | 0 | 0 | 0 |
| 2 | 0 | 10 | 0 | 0 | 0 | 0 | 0 | 0 | 0 | 0 | 0 | 0 | 0 |
| 3 | 0 | 2 | 1 | 0 | 0 | 0 | 0 | 0 | 0 | 0 | 0 | 0 | 0 |
| 4 | 0 | 0 | 0 | 7 | 0 | 0 | 0 | 0 | 0 | 0 | 0 | 0 | 0 |
| 5 | 0 | 0 | 0 | 0 | 2 | 0 | 0 | 0 | 0 | 0 | 0 | 0 | 0 |
| 6 | 0 | 0 | 0 | 0 | 0 | 4 | 1 | 2 | 0 | 0 | 0 | 0 | 0 |
| 7 | 0 | 0 | 0 | 0 | 0 | 0 | 5 | 0 | 0 | 0 | 0 | 0 | 0 |
| 8 | 0 | 0 | 0 | 0 | 0 | 0 | 0 | 10 | 0 | 0 | 0 | 0 | 0 |
| 9 | 0 | 0 | 0 | 0 | 0 | 0 | 1 | 1 | 0 | 0 | 0 | 0 | 0 |
| 10 | 0 | 0 | 0 | 0 | 0 | 0 | 0 | 0 | 0 | 3 | 0 | 0 | 0 |
| 11 | 0 | 0 | 0 | 0 | 0 | 0 | 0 | 2 | 0 | 0 | 0 | 0 | 0 |
| 12 | 0 | 0 | 0 | 0 | 0 | 0 | 0 | 0 | 0 | 0 | 0 | 3 | 0 |
| 13 | 0 | 0 | 0 | 0 | 0 | 0 | 0 | 1 | 0 | 0 | 0 | 0 | 2 |

Table S4 Confusion matrix of PLS-DA in calibration set using mid-level fusion.

| Class | 1 | 2 | 3 | 4 | 5 | 6 | 7 | 8 | 9 | 10 | 11 | 12 | 13 |
| --- | --- | --- | --- | --- | --- | --- | --- | --- | --- | --- | --- | --- | --- |
| 1 | 7 | 0 | 0 | 0 | 0 | 0 | 0 | 0 | 0 | 0 | 0 | 0 | 0 |
| 2 | 0 | 21 | 0 | 0 | 0 | 0 | 0 | 0 | 0 | 0 | 0 | 0 | 0 |
| 3 | 0 | 7 | 0 | 0 | 0 | 0 | 0 | 0 | 0 | 0 | 0 | 0 | 0 |
| 4 | 0 | 2 | 0 | 12 | 0 | 0 | 0 | 0 | 0 | 0 | 0 | 0 | 0 |
| 5 | 0 | 0 | 0 | 0 | 5 | 0 | 0 | 0 | 0 | 0 | 0 | 0 | 0 |
| 6 | 0 | 0 | 0 | 0 | 0 | 12 | 1 | 0 | 0 | 0 | 0 | 0 | 0 |
| 7 | 0 | 0 | 0 | 0 | 0 | 0 | 11 | 0 | 0 | 0 | 0 | 0 | 0 |
| 8 | 0 | 0 | 0 | 0 | 0 | 0 | 0 | 20 | 0 | 0 | 0 | 0 | 0 |
| 9 | 0 | 0 | 0 | 0 | 1 | 0 | 2 | 0 | 0 | 0 | 0 | 0 | 0 |
| 10 | 0 | 0 | 0 | 0 | 0 | 0 | 0 | 0 | 0 | 7 | 0 | 0 | 0 |
| 11 | 0 | 0 | 0 | 0 | 0 | 0 | 0 | 1 | 0 | 0 | 3 | 0 | 0 |
| 12 | 0 | 0 | 0 | 0 | 0 | 0 | 0 | 2 | 0 | 0 | 0 | 5 | 0 |
| 13 | 0 | 0 | 0 | 0 | 0 | 0 | 0 | 0 | 0 | 0 | 0 | 0 | 7 |

Table S5 Confusion matrix of PLS-DA in validation set using mid-level fusion.

| Class | 1 | 2 | 3 | 4 | 5 | 6 | 7 | 8 | 9 | 10 | 11 | 12 | 13 |
| --- | --- | --- | --- | --- | --- | --- | --- | --- | --- | --- | --- | --- | --- |
| 1 | 3 | 1 | 0 | 0 | 0 | 0 | 0 | 0 | 0 | 0 | 0 | 0 | 0 |
| 2 | 0 | 10 | 0 | 0 | 0 | 0 | 0 | 0 | 0 | 0 | 0 | 0 | 0 |
| 3 | 0 | 3 | 0 | 0 | 0 | 0 | 0 | 0 | 0 | 0 | 0 | 0 | 0 |
| 4 | 0 | 0 | 0 | 7 | 0 | 0 | 0 | 0 | 0 | 0 | 0 | 0 | 0 |
| 5 | 0 | 0 | 0 | 0 | 2 | 0 | 0 | 0 | 0 | 0 | 0 | 0 | 0 |
| 6 | 0 | 0 | 0 | 0 | 0 | 4 | 2 | 1 | 0 | 0 | 0 | 0 | 0 |
| 7 | 0 | 0 | 0 | 0 | 0 | 0 | 5 | 0 | 0 | 0 | 0 | 0 | 0 |
| 8 | 0 | 0 | 0 | 0 | 0 | 0 | 0 | 10 | 0 | 0 | 0 | 0 | 0 |
| 9 | 0 | 0 | 0 | 0 | 0 | 0 | 2 | 0 | 0 | 0 | 0 | 0 | 0 |
| 10 | 0 | 0 | 0 | 0 | 0 | 0 | 0 | 0 | 0 | 3 | 0 | 0 | 0 |
| 11 | 0 | 0 | 0 | 0 | 0 | 0 | 0 | 1 | 0 | 1 | 0 | 0 | 0 |
| 12 | 0 | 0 | 0 | 0 | 0 | 0 | 0 | 2 | 0 | 0 | 0 | 1 | 0 |
| 13 | 0 | 0 | 0 | 0 | 0 | 0 | 0 | 1 | 0 | 0 | 0 | 0 | 2 |

Table S6 Confusion matrix of PLS-DA in calibration set using high-level fusion.

| Class | 1 | 2 | 3 | 4 | 5 | 6 | 7 | 8 | 9 | 10 | 11 | 12 | 13 | No class |
| --- | --- | --- | --- | --- | --- | --- | --- | --- | --- | --- | --- | --- | --- | --- |
| 1 | 7 | 0 | 0 | 0 | 0 | 0 | 0 | 0 | 0 | 0 | 0 | 0 | 0 | 0 |
| 2 | 0 | 21 | 0 | 0 | 0 | 0 | 0 | 0 | 0 | 0 | 0 | 0 | 0 | 0 |
| 3 | 0 | 0 | 7 | 0 | 0 | 0 | 0 | 0 | 0 | 0 | 0 | 0 | 0 | 0 |
| 4 | 0 | 0 | 0 | 14 | 0 | 0 | 0 | 0 | 0 | 0 | 0 | 0 | 0 | 0 |
| 5 | 0 | 0 | 0 | 0 | 5 | 0 | 0 | 0 | 0 | 0 | 0 | 0 | 0 | 0 |
| 6 | 0 | 0 | 0 | 0 | 0 | 13 | 1 | 0 | 0 | 0 | 0 | 0 | 0 | 1 |
| 7 | 0 | 0 | 0 | 0 | 0 | 0 | 11 | 0 | 0 | 0 | 0 | 0 | 0 | 0 |
| 8 | 0 | 0 | 0 | 0 | 0 | 0 | 0 | 20 | 0 | 0 | 0 | 0 | 0 | 0 |
| 9 | 0 | 0 | 0 | 0 | 0 | 0 | 0 | 0 | 3 | 0 | 0 | 0 | 0 | 0 |
| 10 | 0 | 0 | 0 | 0 | 0 | 0 | 0 | 0 | 0 | 7 | 0 | 0 | 0 | 0 |
| 11 | 0 | 0 | 0 | 0 | 0 | 0 | 0 | 0 | 0 | 0 | 4 | 0 | 0 | 0 |
| 12 | 0 | 0 | 0 | 0 | 0 | 0 | 0 | 0 | 0 | 0 | 0 | 7 | 0 | 0 |
| 13 | 0 | 0 | 0 | 0 | 0 | 0 | 0 | 0 | 0 | 0 | 0 | 0 | 7 | 0 |
| SEN (%) | 100.00 | 100.00 | 100.00 | 100.00 | 100.00 | 100.00 | 100.00 | 100.00 | 100.00 | 100.00 | 100.00 | 100.00 | 100.00 | － |
| SPE (%) | 100.00 | 100.00 | 100.00 | 100.00 | 100.00 | 100.00 | 100.00 | 100.00 | 100.00 | 100.00 | 100.00 | 100.00 | 100.00 | － |
| PRE (%) | 100.00 | 100.00 | 100.00 | 100.00 | 100.00 | 100.00 | 100.00 | 100.00 | 100.00 | 100.00 | 100.00 | 100.00 | 100.00 | － |
| EFF (%) | 100.00 | 100.00 | 100.00 | 100.00 | 100.00 | 100.00 | 100.00 | 100.00 | 100.00 | 100.00 | 100.00 | 100.00 | 100.00 | － |

－: Not be calculated

Table S7 Confusion matrix of PLS-DA in validation set using high-level fusion.

|  | 1 | 2 | 3 | 4 | 5 | 6 | 7 | 8 | 9 | 10 | 11 | 12 | 13 | No class |
| --- | --- | --- | --- | --- | --- | --- | --- | --- | --- | --- | --- | --- | --- | --- |
| 1 | 4 | 0 | 0 | 0 | 0 | 0 | 0 | 0 | 0 | 0 | 0 | 0 | 0 | 0 |
| 2 | 0 | 10 | 0 | 0 | 0 | 0 | 0 | 0 | 0 | 0 | 0 | 0 | 0 | 0 |
| 3 | 0 | 2 | 2 | 0 | 0 | 0 | 0 | 0 | 0 | 0 | 0 | 0 | 0 | 1 |
| 4 | 0 | 0 | 0 | 7 | 0 | 0 | 0 | 0 | 0 | 0 | 0 | 0 | 0 | 0 |
| 5 | 0 | 0 | 0 | 0 | 2 | 0 | 0 | 0 | 0 | 0 | 0 | 0 | 0 | 0 |
| 6 | 0 | 2 | 0 | 0 | 0 | 2 | 2 | 2 | 0 | 0 | 0 | 0 | 0 | 1 |
| 7 | 0 | 0 | 0 | 0 | 0 | 0 | 5 | 0 | 0 | 0 | 0 | 0 | 0 | 0 |
| 8 | 0 | 0 | 0 | 0 | 0 | 0 | 0 | 10 | 0 | 0 | 0 | 0 | 0 | 0 |
| 9 | 0 | 0 | 0 | 0 | 0 | 0 | 1 | 1 | 1 | 0 | 0 | 0 | 0 | 1 |
| 10 | 0 | 0 | 0 | 0 | 0 | 0 | 0 | 0 | 0 | 3 | 0 | 0 | 0 | 0 |
| 11 | 0 | 0 | 0 | 0 | 0 | 0 | 0 | 1 | 0 | 0 | 1 | 0 | 1 | 1 |
| 12 | 0 | 0 | 0 | 0 | 0 | 0 | 0 | 0 | 0 | 0 | 0 | 3 | 0 | 0 |
| 13 | 0 | 0 | 0 | 0 | 0 | 0 | 0 | 0 | 0 | 0 | 0 | 0 | 3 | 0 |
| SEN (%) | 100.00 | 100.00 | 66.67 | 100.00 | 100.00 | 66.67 | 100.00 | 100.00 | 50.00 | 100.00 | 50.00 | 100.00 | 100.00 | － |
| SPE (%) | 100.00 | 96.08 | 100.00 | 100.00 | 100.00 | 100.00 | 95.16 | 94.12 | 100.00 | 100.00 | 100.00 | 100.00 | 100.00 | － |
| PRE (%) | 100.00 | 83.33 | 100.00 | 100.00 | 100.00 | 100.00 | 62.50 | 76.92 | 100.00 | 100.00 | 100.00 | 100.00 | 100.00 | － |
| EFF (%) | 100.00 | 98.02 | 81.65 | 100.00 | 100.00 | 81.65 | 97.55 | 97.01 | 70.71 | 100.00 | 70.71 | 100.00 | 100.00 | － |

－: Not be calculated

Table S8 Confusion matrix of RF in calibration set using low-level fusion.

|  | 1 | 2 | 3 | 4 | 5 | 6 | 7 | 8 | 9 | 10 | 11 | 12 | 13 |
| --- | --- | --- | --- | --- | --- | --- | --- | --- | --- | --- | --- | --- | --- |
| 1 | 7 | 0 | 0 | 0 | 0 | 0 | 0 | 0 | 0 | 0 | 0 | 0 | 0 |
| 2 | 0 | 21 | 0 | 0 | 0 | 0 | 0 | 0 | 0 | 0 | 0 | 0 | 0 |
| 3 | 0 | 0 | 3 | 4 | 0 | 0 | 0 | 0 | 0 | 0 | 0 | 0 | 0 |
| 4 | 0 | 0 | 0 | 14 | 0 | 0 | 0 | 0 | 0 | 0 | 0 | 0 | 0 |
| 5 | 0 | 0 | 0 | 0 | 4 | 0 | 1 | 0 | 0 | 0 | 0 | 0 | 0 |
| 6 | 0 | 0 | 0 | 0 | 0 | 10 | 3 | 0 | 0 | 0 | 0 | 0 | 0 |
| 7 | 0 | 0 | 0 | 0 | 0 | 0 | 9 | 2 | 0 | 0 | 0 | 0 | 0 |
| 8 | 0 | 0 | 0 | 0 | 0 | 0 | 0 | 20 | 0 | 0 | 0 | 0 | 0 |
| 9 | 0 | 0 | 0 | 0 | 0 | 0 | 1 | 2 | 0 | 0 | 0 | 0 | 0 |
| 10 | 0 | 0 | 0 | 0 | 0 | 0 | 0 | 0 | 0 | 7 | 0 | 0 | 0 |
| 11 | 0 | 0 | 0 | 0 | 0 | 0 | 0 | 2 | 0 | 0 | 0 | 0 | 2 |
| 12 | 0 | 0 | 0 | 0 | 0 | 0 | 0 | 1 | 0 | 0 | 0 | 6 | 0 |
| 13 | 0 | 0 | 0 | 0 | 0 | 0 | 0 | 0 | 0 | 0 | 0 | 0 | 7 |
| SEN (%) | 100.00 | 100.00 | 42.86 | 100.00 | 80.00 | 76.92 | 81.82 | 100.00 | 0 | 100.00 | 0 | 85.71 | 100.00 |
| SPE (%) | 100.00 | 100.00 | 100.00 | 96.43 | 100.00 | 100.00 | 95.73 | 93.40 | 100.00 | 100.00 | 100.00 | 100.00 | 98.32 |
| PRE (%) | 100.00 | 100.00 | 100.00 | 77.78 | 100.00 | 100.00 | 64.29 | 74.07 | － | 100.00 | － | 100.00 | 77.78 |
| EFF (%) | 100.00 | 100.00 | 65.47 | 98.20 | 89.44 | 87.71 | 88.50 | 96.64 | 0 | 100.00 | 0 | 92.58 | 99.16 |

－: Not be calculated

Table S9 Confusion matrix of RF in validation set using low-level fusion.

|  | 1 | 2 | 3 | 4 | 5 | 6 | 7 | 8 | 9 | 10 | 11 | 12 | 13 |
| --- | --- | --- | --- | --- | --- | --- | --- | --- | --- | --- | --- | --- | --- |
| 1 | 4 | 0 | 0 | 0 | 0 | 0 | 0 | 0 | 0 | 0 | 0 | 0 | 0 |
| 2 | 0 | 10 | 0 | 0 | 0 | 0 | 0 | 0 | 0 | 0 | 0 | 0 | 0 |
| 3 | 0 | 1 | 2 | 0 | 0 | 0 | 0 | 0 | 0 | 0 | 0 | 0 | 0 |
| 4 | 0 | 0 | 0 | 7 | 0 | 0 | 0 | 0 | 0 | 0 | 0 | 0 | 0 |
| 5 | 0 | 0 | 0 | 0 | 1 | 0 | 1 | 0 | 0 | 0 | 0 | 0 | 0 |
| 6 | 0 | 0 | 0 | 0 | 0 | 1 | 6 | 0 | 0 | 0 | 0 | 0 | 0 |
| 7 | 0 | 0 | 0 | 0 | 0 | 0 | 5 | 0 | 0 | 0 | 0 | 0 | 0 |
| 8 | 0 | 0 | 0 | 0 | 0 | 0 | 0 | 10 | 0 | 0 | 0 | 0 | 0 |
| 9 | 0 | 0 | 0 | 0 | 0 | 0 | 0 | 0 | 2 | 0 | 0 | 0 | 0 |
| 10 | 0 | 0 | 0 | 0 | 0 | 0 | 0 | 0 | 0 | 3 | 0 | 0 | 0 |
| 11 | 0 | 0 | 0 | 0 | 0 | 0 | 0 | 1 | 0 | 0 | 0 | 0 | 1 |
| 12 | 0 | 0 | 0 | 0 | 0 | 0 | 0 | 0 | 0 | 0 | 0 | 3 | 0 |
| 13 | 0 | 0 | 0 | 0 | 0 | 0 | 0 | 0 | 0 | 0 | 0 | 0 | 3 |
| SEN (%) | 100.00 | 100.00 | 66.67 | 100.00 | 50.00 | 14.29 | 100.00 | 100.00 | 100.00 | 100.00 | 0 | 100.00 | 100.00 |
| SPE (%) | 100.00 | 98.04 | 100.00 | 100.00 | 100.00 | 100.00 | 87.50 | 98.04 | 100.00 | 100.00 | 100.00 | 100.00 | 98.28 |
| PRE (%) | 100.00 | 90.91 | 100.00 | 100.00 | 100.00 | 100.00 | 41.67 | 90.91 | 100.00 | 100.00 | － | 100.00 | 75.00 |
| EFF (%) | 100.00 | 99.01 | 81.65 | 100.00 | 70.71 | 37.80 | 93.54 | 99.01 | 100.00 | 100.00 | 0 | 100.00 | 99.13 |

－: Not be calculated

Table S10 Confusion matrix of RF in calibration set using mid-level fusion.

|  | 1 | 2 | 3 | 4 | 5 | 6 | 7 | 8 | 9 | 10 | 11 | 12 | 13 |
| --- | --- | --- | --- | --- | --- | --- | --- | --- | --- | --- | --- | --- | --- |
| 1 | 7 | 0 | 0 | 0 | 0 | 0 | 0 | 0 | 0 | 0 | 0 | 0 | 0 |
| 2 | 0 | 21 | 0 | 0 | 0 | 0 | 0 | 0 | 0 | 0 | 0 | 0 | 0 |
| 3 | 0 | 3 | 3 | 1 | 0 | 0 | 0 | 0 | 0 | 0 | 0 | 0 | 0 |
| 4 | 0 | 0 | 0 | 14 | 0 | 0 | 0 | 0 | 0 | 0 | 0 | 0 | 0 |
| 5 | 0 | 0 | 0 | 1 | 3 | 0 | 1 | 0 | 0 | 0 | 0 | 0 | 0 |
| 6 | 0 | 1 | 0 | 0 | 0 | 10 | 1 | 1 | 0 | 0 | 0 | 0 | 0 |
| 7 | 0 | 1 | 0 | 0 | 0 | 0 | 8 | 2 | 0 | 0 | 0 | 0 | 0 |
| 8 | 0 | 0 | 0 | 0 | 0 | 0 | 0 | 20 | 0 | 0 | 0 | 0 | 0 |
| 9 | 0 | 0 | 0 | 0 | 0 | 0 | 1 | 1 | 0 | 0 | 0 | 0 | 1 |
| 10 | 0 | 0 | 0 | 0 | 0 | 0 | 0 | 0 | 0 | 7 | 0 | 0 | 0 |
| 11 | 0 | 0 | 0 | 0 | 0 | 0 | 0 | 3 | 0 | 0 | 0 | 0 | 1 |
| 12 | 0 | 1 | 0 | 0 | 0 | 2 | 0 | 1 | 0 | 0 | 0 | 3 | 0 |
| 13 | 0 | 0 | 0 | 0 | 0 | 0 | 0 | 0 | 0 | 0 | 0 | 0 | 7 |
| SEN (%) | 100.00 | 100.00 | 42.86 | 100.00 | 60.00 | 76.92 | 72.73 | 100.00 | 0 | 100.00 | 0 | 42.86 | 100.00 |
| SPE (%) | 100.00 | 94.29 | 100.00 | 98.21 | 100.00 | 98.28 | 97.46 | 92.45 | 100.00 | 100.00 | 100.00 | 100.00 | 98.32 |
| PRE (%) | 100.00 | 77.78 | 100.00 | 87.50 | 100.00 | 83.33 | 72.73 | 71.43 | － | 100.00 | － | 100.00 | 77.78 |
| EFF (%) | 100.00 | 97.10 | 65.47 | 99.10 | 77.46 | 86.95 | 84.19 | 96.15 | 0 | 100.00 | 0 | 65.47 | 99.16 |

－: Not be calculated

Table S11 Confusion matrix of RF in validation set using mid-level fusion.

|  | 1 | 2 | 3 | 4 | 5 | 6 | 7 | 8 | 9 | 10 | 11 | 12 | 13 |
| --- | --- | --- | --- | --- | --- | --- | --- | --- | --- | --- | --- | --- | --- |
| 1 | 4 | 0 | 0 | 0 | 0 | 0 | 0 | 0 | 0 | 0 | 0 | 0 | 0 |
| 2 | 0 | 10 | 0 | 0 | 0 | 0 | 0 | 0 | 0 | 0 | 0 | 0 | 0 |
| 3 | 0 | 1 | 1 | 1 | 0 | 0 | 0 | 0 | 0 | 0 | 0 | 0 | 0 |
| 4 | 0 | 0 | 0 | 7 | 0 | 0 | 0 | 0 | 0 | 0 | 0 | 0 | 0 |
| 5 | 0 | 0 | 0 | 0 | 2 | 0 | 0 | 0 | 0 | 0 | 0 | 0 | 0 |
| 6 | 0 | 0 | 0 | 0 | 0 | 4 | 1 | 2 | 0 | 0 | 0 | 0 | 0 |
| 7 | 0 | 0 | 0 | 0 | 0 | 0 | 5 | 0 | 0 | 0 | 0 | 0 | 0 |
| 8 | 0 | 0 | 0 | 0 | 0 | 0 | 0 | 10 | 0 | 0 | 0 | 0 | 0 |
| 9 | 0 | 0 | 0 | 0 | 0 | 0 | 1 | 1 | 0 | 0 | 0 | 0 | 0 |
| 10 | 0 | 0 | 0 | 0 | 0 | 0 | 0 | 0 | 0 | 3 | 0 | 0 | 0 |
| 11 | 0 | 0 | 0 | 0 | 0 | 0 | 0 | 0 | 0 | 0 | 2 | 0 | 0 |
| 12 | 0 | 0 | 0 | 0 | 0 | 0 | 0 | 0 | 0 | 0 | 0 | 3 | 0 |
| 13 | 0 | 0 | 0 | 0 | 0 | 0 | 0 | 0 | 0 | 0 | 0 | 0 | 3 |
| SEN (%) | 100.00 | 100.00 | 33.33 | 100.00 | 100.00 | 57.14 | 100.00 | 100.00 | 0 | 100.00 | 100.00 | 100.00 | 100.00 |
| SPE (%) | 100.00 | 98.04 | 100.00 | 98.15 | 100.00 | 100.00 | 96.43 | 94.12 | 100.00 | 100.00 | 100.00 | 100.00 | 100.00 |
| PRE (%) | 100.00 | 90.91 | 100.00 | 87.50 | 100.00 | 100.00 | 71.43 | 76.92 | － | 100.00 | 100.00 | 100.00 | 100.00 |
| EFF (%) | 100.00 | 99.01 | 57.74 | 99.07 | 100.00 | 75.59 | 98.20 | 97.01 | 0 | 100.00 | 100.00 | 100.00 | 100.00 |

－: Not be calculated

Table S12 Confusion matrix of RF in calibration set using high-level fusion.

| Class | 1 | 2 | 3 | 4 | 5 | 6 | 7 | 8 | 9 | 10 | 11 | 12 | 13 | No class |
| --- | --- | --- | --- | --- | --- | --- | --- | --- | --- | --- | --- | --- | --- | --- |
| 1 | 7 | 0 | 0 | 0 | 0 | 0 | 0 | 0 | 0 | 0 | 0 | 0 | 0 | 0 |
| 2 | 0 | 21 | 0 | 0 | 0 | 0 | 0 | 0 | 0 | 0 | 0 | 0 | 0 | 0 |
| 3 | 0 | 0 | 6 | 1 | 0 | 0 | 0 | 0 | 0 | 0 | 0 | 0 | 0 | 0 |
| 4 | 0 | 0 | 0 | 14 | 0 | 0 | 0 | 0 | 0 | 0 | 0 | 0 | 0 | 0 |
| 5 | 0 | 0 | 0 | 0 | 5 | 0 | 1 | 0 | 0 | 0 | 0 | 0 | 0 | 1 |
| 6 | 0 | 0 | 0 | 0 | 0 | 11 | 3 | 1 | 0 | 0 | 0 | 0 | 0 | 3 |
| 7 | 0 | 0 | 0 | 1 | 0 | 0 | 10 | 2 | 0 | 0 | 0 | 0 | 0 | 2 |
| 8 | 0 | 0 | 0 | 0 | 0 | 0 | 0 | 20 | 0 | 0 | 0 | 0 | 0 | 0 |
| 9 | 0 | 0 | 0 | 0 | 0 | 0 | 1 | 3 | 0 | 0 | 0 | 0 | 0 | 1 |
| 10 | 0 | 0 | 0 | 0 | 0 | 0 | 0 | 0 | 0 | 7 | 0 | 0 | 0 | 0 |
| 11 | 0 | 0 | 0 | 0 | 0 | 0 | 0 | 1 | 0 | 0 | 2 | 0 | 1 | 0 |
| 12 | 0 | 0 | 0 | 0 | 0 | 0 | 0 | 1 | 0 | 0 | 0 | 7 | 0 | 1 |
| 13 | 0 | 0 | 0 | 0 | 0 | 0 | 0 | 0 | 0 | 0 | 0 | 0 | 7 | 0 |
| SEN (%) | 100.00 | 100.00 | 85.71 | 100.00 | 100.00 | 78.57 | 90.91 | 100.00 | 0 | 100.00 | 66.67 | 100.00 | 100.00 | － |
| SPE (%) | 100.00 | 100.00 | 100.00 | 99.11 | 100.00 | 100.00 | 96.55 | 92.45 | 100.00 | 100.00 | 100.00 | 100.00 | 99.16 | － |
| PRE (%) | 100.00 | 100.00 | 100.00 | 93.33 | 100.00 | 100.00 | 71.43 | 71.43 | － | 100.00 | 100.00 | 100.00 | 87.50 | － |
| EFF (%) | 100.00 | 100.00 | 92.58 | 99.55 | 100.00 | 88.64 | 93.69 | 96.15 | 0 | 100.00 | 81.65 | 100.00 | 99.58 | － |

－: Not be calculated

Table S13 Confusion matrix of RF in validation set using high-level fusion.

| Class | 1 | 2 | 3 | 4 | 5 | 6 | 7 | 8 | 9 | 10 | 11 | 12 | 13 | No class |
| --- | --- | --- | --- | --- | --- | --- | --- | --- | --- | --- | --- | --- | --- | --- |
| 1 | 4 | 0 | 0 | 0 | 0 | 0 | 0 | 0 | 0 | 0 | 0 | 0 | 0 | 0 |
| 2 | 0 | 10 | 0 | 0 | 0 | 0 | 0 | 0 | 0 | 0 | 0 | 0 | 0 | 0 |
| 3 | 0 | 0 | 3 | 0 | 0 | 0 | 0 | 0 | 0 | 0 | 0 | 0 | 0 | 0 |
| 4 | 0 | 0 | 0 | 7 | 0 | 0 | 0 | 0 | 0 | 0 | 0 | 0 | 0 | 0 |
| 5 | 0 | 0 | 0 | 0 | 2 | 0 | 0 | 0 | 0 | 0 | 0 | 0 | 0 | 0 |
| 6 | 0 | 0 | 0 | 0 | 0 | 4 | 4 | 1 | 0 | 0 | 0 | 0 | 0 | 2 |
| 7 | 0 | 0 | 0 | 0 | 0 | 0 | 5 | 0 | 0 | 0 | 0 | 0 | 0 | 0 |
| 8 | 0 | 0 | 0 | 0 | 0 | 0 | 0 | 10 | 0 | 0 | 0 | 0 | 0 | 0 |
| 9 | 0 | 0 | 0 | 0 | 0 | 0 | 0 | 0 | 2 | 0 | 0 | 0 | 0 | 0 |
| 10 | 0 | 0 | 0 | 0 | 0 | 0 | 0 | 0 | 0 | 3 | 0 | 0 | 0 | 0 |
| 11 | 0 | 0 | 0 | 0 | 0 | 0 | 0 | 1 | 0 | 0 | 1 | 0 | 1 | 1 |
| 12 | 0 | 0 | 0 | 0 | 0 | 0 | 0 | 0 | 0 | 0 | 0 | 3 | 0 | 0 |
| 13 | 0 | 0 | 0 | 0 | 0 | 0 | 0 | 0 | 0 | 0 | 0 | 0 | 3 | 0 |
| SEN (%) | 100.00 | 100.00 | 100.00 | 100.00 | 100.00 | 57.14 | 100.00 | 100.00 | 100.00 | 100.00 | 50.00 | 100.00 | 100.00 | － |
| SPE (%) | 100.00 | 100.00 | 100.00 | 100.00 | 100.00 | 100.00 | 96.43 | 98.04 | 100.00 | 100.00 | 100.00 | 100.00 | 98.28 | － |
| PRE (%) | 100.00 | 100.00 | 100.00 | 100.00 | 100.00 | 100.00 | 71.43 | 90.91 | 100.00 | 100.00 | 100.00 | 100.00 | 75.00 | － |
| EFF (%) | 100.00 | 100.00 | 100.00 | 100.00 | 100.00 | 75.59 | 98.20 | 99.01 | 100.00 | 100.00 | 70.71 | 100.00 | 99.13 | － |

－: Not be calculated
